# Disentangling Heterogeneity in the Co‐Developmental of Anxiety and Depression During the COVID‐19 Pandemic: Insights From Cross‐Lagged Panel Network

**DOI:** 10.1155/da/6008342

**Published:** 2026-02-23

**Authors:** Zijuan Ma, Xiao-Yan Chen, Zhijun Yu, Yang Li, Shaochen Zhao, Dongfang Wang, Fang Fan

**Affiliations:** ^1^ Center for Studies of Psychological Application, School of Psychology, and Guangdong Key Laboratory of Mental Health and Cognitive Science, South China Normal University, Guangzhou, China, scnu.edu.cn; ^2^ Department of Educational Psychology, Faculty of Education, The Chinese University of Hong Kong, Hong Kong, China, cuhk.edu.hk; ^3^ Research Center for Guangdong-HongKong-Marcao Policing Model Innovation, China People’s Police University, Guangzhou, China

**Keywords:** anxiety, co-developmental trajectories, comorbidity rates, depression, dynamics network

## Abstract

**Background:**

Previous studies have indicated substantial variations in anxiety and depressive symptoms over the course of the COVID‐19 pandemic. However, little is known about the comorbidity rates, heterogeneous co‐developmental trajectories, and temporal network dynamics of these symptoms. Integrating person‐centered and network psychopathology approaches, this study aimed to investigate the comorbidity rates and longitudinal network structures of anxiety and depressive symptoms across distinct co‐developmental trajectories during the pandemic.

**Methods:**

A total of 35,516 college students completed three‐wave surveys during the COVID‐19 pandemic. Anxiety and depressive symptoms were assessed using the Generalized Anxiety Disorder‐7‐item and the 9‐item Patient Health Questionnaire, respectively. Co‐developmental trajectories were identified via a Parallel‐Process Latent Growth Curve Model, and symptom networks were constructed using a cross‐lagged panel network (CLPN).

**Results:**

Results showed that over 40% of individuals with depression also experienced anxiety across different pandemic phases. Three heterogeneous co‐developmental trajectories were identified: a resistance group, a persistent growth group, and a chronic co‐occurring group. The most influential symptoms—those exerting the strongest effect on subsequent symptoms—varied across the three networks. In the resistance group, “Anhedonia” was the most central symptom; in the persistent growth group, “Motor” exhibited the strongest predictive effect; and in the chronic co‐occurring group, “Guilt” was the most influential symptom.

**Conclusions:**

These findings highlight the necessity of developing targeted and individualized interventions tailored to specific symptom dynamics in different youth subgroups to alleviate the overall symptom burden in future public health crises.

## 1. Introduction

The global spread of coronavirus disease 2019 (COVID‐19) has not only threatened physical health but also contributed to various psychological problems, mainly due to the disruptions and restrictions introduced by the COVID‐19 pandemic [1, 2]. Recent meta‐analyses indicate that the overall prevalence of anxiety and depressive symptoms among university students was 45% and 43%–48% during the COVID‐19 pandemic, respectively [2, 3]. Furthermore, a meta‐analysis with 53 longitudinal studies has found an increase in these symptoms among children and adolescents compared to prepandemic levels [4]. Notably, anxiety and depression are known to co‐occur frequently [5–8], and this comorbidity appears to have been intensified throughout the COVID‐19 pandemic. This exacerbation may be attributed to the COVID‐19 impact on daily function, such as impairing individuals’ ability to work or study, reducing physical activity, heightening fatigue, and compounding other health concerns [9]. Despite these insights, the proportion, patterns, and mechanisms underlying the comorbidity of anxiety and depression during the pandemic remain poorly understood.

During the COVID‐19 pandemic, anxiety and depressive symptoms have exhibited considerable variation across populations over time [10]. Several studies have adopted person‐centered methods (i.e., latent growth curve models, LGCM; and growth mixture models, GMM) using multi‐wave longitudinal data to identify symptom trajectories of anxiety and depression. These investigations commonly identified three to four trajectories for anxiety or depression [11, 12]. A study, applying a cutoff point of clinical anxiety and depression across three pandemic phases, further delineated five anxiety trajectories (resistance: 72.7%; recovery: 4.9%; delayed‐dysfunction: 11.8%; relapsing/remitting: 6.1%; and chronic‐dysfunction: 4.5%) and five depression trajectories (resistance: 55.8%; recovery: 6.6%; delayed‐dysfunction: 17.7%; relapsing/remitting: 8.4%; and chronic‐dysfunction: 11.6%;) [13]. While these studies successfully identified discrete symptom pathways using LGCM or GMM, their focus on modeling each outcome separately may limit our understanding of the interrelated nature of dimensional constructs like anxiety and depression. Notably, long‐term COVID‐19 has emerged as only a significant correlate of probable comorbid depression and anxiety [9], underscoring the need to examine how these conditions dynamically co‐evolve over time. The Parallel‐Process Latent Growth Curve Model (PP‐LGCM) provides a robust analytical framework for assessing how initial level (intercepts) and longitudinal changes (slopes) of anxiety and depression interrelate [14]. Applying this model, this study aims to move beyond isolated trajectory analyses and toward a more integrated understanding of how anxiety and depressive symptoms co‐develop across three key pandemic phases: the initial outbreak, initial remission, and control phases.

Research on mental health during the COVID‐19 pandemic has increasingly sought to identify the mechanisms underlying the high prevalence and comorbidity of depression and anxiety to improve detection and intervention strategies [1]. In this context, network theory has emerged as a novel and valuable framework for understanding psychopathology [15, 16]. Based on this theory, network analysis has been developed to conceptualize symptoms as constitutive elements of mental illnesses and to construct a complex network of mutually interacting symptoms [17, 18]. Although several studies have employed network analysis to examine the structure of anxiety and depression during COVID‐19 [19–22], most rely on cross‐sectional data, which cannot establish temporal precedence among symptoms. Longitudinal network modeling is essential for capturing how symptom systems evolve [23]. To address this, the cross‐lagged panel network (CLPN) method has been developed, enabling the analysis of dynamic, reciprocal relationships among multiple variables across two time points [24, 25]. To date, only one study has used CLPN to explore the dynamic interplay between anxiety, depression, and cognitive functioning components [26]. However, it remains unclear whether such networks are replicable across different trajectories during different pandemic phases. Therefore, this study is also to conceptualize the symptom systems of anxiety and depression across the heterogeneous co‐developmental trajectories during the COVID‐19 pandemic.

## 2. Methods

### 2.1. Study Cohort and Participants

Data were derived from the College Students’ Behavior and Health Cohort during the COVID‐19 pandemic (CSBHC_COVID‐19). Employing a repeated cross‐sectional design, this project collected data via online surveys administered at 22 colleges and universities across three pandemic phases. The initial survey (Time 1, T1) was conducted during the outbreak peak (February 3–10, 2020) with 185,901 participants. This was followed by a second survey during the initial remission period (Time 2, T2: March 24 to April 3, 2020; *N* = 155,563) and a third during the control period (Time 3, T3: June 1–15, 2020; *N* = 166,052), respectively. These three phases were strategically selected for their unique epidemiological characteristics, societal responses, and psychological impacts [27], which collectively capture acute reactions and critical shifts during the pandemic.

A total of 35,516 students completed all three surveys successfully. More detailed sampling information is shown in Table S1. Online Figure S1 displays the trend of the COVID‐19 pandemic in China. Figure S2 illustrates the geographical distribution of participating colleges/universities. Details of sampling and data collection have been described previously [13, 28, 29]. Prior to the administration of surveys, electronic informed consent was obtained from each participant or their legal guardians (under the age of 18). In addition, participants were explicitly informed that their involvement was entirely voluntary and that they had the right to withdraw from the study at any time without penalty. This study was carried out in accordance with the Helsinki Declaration and approved by the Human Research Ethics Committee of South China Normal University (Ethics Number: SCNUPSY‐2020‐01‐001).

### 2.2. Measures

Data on demographic characteristics (sex and age) and pandemic‐related factors were collected at T1. Several pandemic‐related factors included the provincial‐level epidemic severity, the presence of confirmed or suspected cases in the community or village, relatives or acquaintances infected with COVID‐19, and the extent of exposure to COVID‐19 media coverage.

Anxiety and depressive symptoms in the past 2 weeks were assessed at T1, T2, and T3 using the 7‐item Generalized Anxiety Disorder Scale (GAD‐7) and the 9‐item Patient Health Questionnaire (PHQ‐9), respectively [30, 31]. Both the Chinese versions of the GAD‐7 and PHQ‐9 have demonstrated strong psychometric properties as screening tools for anxiety and depressive symptoms [32, 33]. Items were rated on a 4‐point Likert scale (0 = *“not at all”* to 3 = *“nearly every day”*), with higher scores indicating greater severity. A total score of ≥7 was adopted as the cutoff point for clinical‐level symptoms [32]. To estimate the likelihood (odds ratio, ORs) that a symptom’s presence at T1 predicts its own or another symptom’s occurrence at T2 [34], we transformed the item scores into a binary format (0 = absence; 1–3 = presence). Both scales demonstrated high internal consistency across all waves (GAD‐7: α_T1_ = 0.91, α_T2_ = 0.93, α_T3_ = 0.94; PHQ‐9: α_T1_ = 0.87, α_T2_ = 0.90, α_T3_ = 0.91).

### 2.3. Statistical Analyses

Descriptive statistics and correlation analysis were conducted with SPSS 25.0. The heterogeneous co‐developmental trajectories and CLPNs were analyzed using Mplus 7.4 and R 4.1.3, respectively.

### 2.4. The Heterogeneous Co‐Developmental Trajectories

The primary analyses examining the heterogeneous co‐developmental trajectories of anxiety and depressive symptoms consisted of four steps. First, Pearson correlation was conducted to assess the relationship between anxiety and depression. Second, confirmatory factor analysis (CFA) was used to evaluate the longitudinal measurement invariance of the GAD‐7 and PHQ‐9 across the three waves [35]. Specifically, three levels of invariance were tested: configural, metric, and scalar invariance. In line with established studies [36], a decrease in the comparative fit index (△CFI) of less than 0.01 was considered indicative of invariant measurements. Third, the PP‐LGCM was specified to examine the co‐developmental trajectories for anxiety and depressive symptoms. A linear growth form was adopted due to the limited number of measurement time points (*n* = 3). Mean scores were used in this model to account for the different total scores of the GAD‐7 AND PHQ‐9. Fourth, a series of latent class growth models with 1–5 classes was estimated to identify distinct trajectory subgroups. The optimal model was selected based on the following criteria [14]: (1) lower values on information fit indices, including Akaike Information Criteria (AIC), *Bayesian* Information Criterion (BIC), and sample size adjusted BIC (ABIC); (2) significant *p* ‐values (<0.001) for both the Lo–Mendell–Rubin likelihood ratio test (LMR‐LRT) and bootstrap likelihood ratio tests (BLRT); (3) entropy values greater than 0.80; (4) adequate sample sizes in each class. Theoretical relevance and clinical interpretability were also considered in determining the final model [37].

### 2.5. The CLPN Models

All networks were visualized using the *Fruchterman–Reingold* algorithm in the R‐package *qgraph*. In these graphs, nodes represent individual symptoms, and edges depict relationships between them. The thickness of edges is proportional to the strength of the connection between two symptoms [38, 39].

#### 2.5.1. CLPN

The CLPN model was developed by using regularized regression to estimate both autoregressive and cross‐lagged coefficients. Autoregressive coefficients represent the influence of a variable on itself at the next time point. Cross‐lagged coefficients reflect the predictive effect of one variable on another at the subsequent measurement [25]. In this study, two CLPN models (T1→T2 and T2→T3) were estimated separately. Covariates included relevant demographic and COVID‐19‐related factors at T1 (see Table S1). To enhance interpretability, the logistic regression coefficients (i.e., edge weights) were converted from log odds to ORs [24]. Consequently, an OR greater than 1 signifies a positive relationship, an OR less than one indicates a negative relationship, and an OR equal to 1 indicates no relationship.

#### 2.5.2. Network Inference

We assessed node influence by calculating two centrality indices using the R‐package *qgraph* [38]: in expected influence (in‐EI) and out expected influence (out‐EI). These indices represent the summed strength of all incoming and outgoing connections for a given symptom, respectively [24, 34]. Notably, symptoms with high out‐EI are considered promising intervention targets, as prior longitudinal evidence suggests that influencing them can lead to the alleviation of co‐occurring symptoms [17, 40]. To identify a manageable number of core symptoms for clinical interpretation, we defined a symptom as influential when the out‐EI exceeded one. This threshold, employed in previous network studies (e.g., [41]), denotes a connection strength of substantive practical significance.

#### 2.5.3. Network Accuracy and Stability

The accuracy and stability of all networks were assessed using the *bootstrapping* function in the R‐package *boonet* [42]. Nonparametric bootstrapping with 1000 iterations was employed to evaluate edge‐weight accuracy and to test for the significant differences between edges, with results summarized using 95% confidence intervals (CIs). For centrality stability, the correlation stability coefficient (CS‐C) was computed for in‐EI and out‐EI via a case‐dropping bootstrap procedure. The CS‐C represents the maximum proportion of cases that can be dropped while maintaining a correlation above 0.7 (at the 95% confidence level) between the centrality indices of the original and subsetted samples. The CS‐C values ≥0.25 were deemed acceptable, and values ≥0.50 were considered to indicate good stability [42].

#### 2.5.4. Network Replicability

To examine changes in anxiety‐depression symptom associations across distinct COVID‐19 phases, we compared the T1→T2 and T2→T3 CLPN models. The comparison was based on three aspects: (1) the correlation of edge weights, which offers a global assessment of network similarity; (2) the percentage of edges that were replicated in both models; and (3) the correlation of centrality indices between the two models [24, 43].

## 3. Results

### 3.1. The Comorbidity Rates of Anxiety and Depression During the COVID‐19 Pandemic

The prevalence of anxiety was 11.5% (*n* = 4,077) at T1, 14.4% (*n* = 5,118) at T2, and 18.3% (*n* = 6,502) at T3 (*χ* ^2^ = 663.4, *p* < 0.001, *η* = 0.079). Meanwhile, the prevalence of depression increased from 21.7% (*n* = 7,723) at T1 to 26.2% (*n* = 9,297) at T2, and further to 32.9% (*n* = 11,669) at T3 (*χ* ^2^ = 1129.3, *p* < 0.001, *η* = 0.103). Regarding comorbidity, among individuals with depression at T1, 44.0% (*n* = 3,400) reported anxiety at T1, which decreased to 39.6% (*n* = 3,055) at T2 and then rose to 44.7% (*n* = 3,455) at T3. Of those with depression at T2, 49.3% (*n* = 4,587) reported anxiety at T2, declining to 46.6% (*n* = 4,330) at T3. Among individuals with depression at T3, 52.5% (*n* = 6,125) also reported anxiety at T3.

### 3.2. The Heterogeneous Co‐Developmental Trajectories

Table S2 presents the means, standard deviations (*SD*), and correlations of the total scores for GAD‐7 and PHQ‐9 across the three time points. According to Table S3, CFA supported longitudinal measurement invariance for each scale, meeting the scalar‐level criterion. A PP‐LGCM indicated that a linear trajectory fit the data well (CFI = 0.999, TLI = 0.996, RMSEA = 0.035). Figure 1A illustrates the co‐developmental trajectories of anxiety and depressive symptoms based on mean scores. Furthermore, the intercepts (*r* = 0.101, *p* < 0.001) and slopes (*r* = 0.011, *p* < 0.001) of these trajectories showed significant positive correlations. The variances of both the intercept (anxiety: 0.111, *p* < 0.001; depression: 0.117, *p* < 0.001) and the slope (anxiety: 0.014, *p* < 0.001; depression: 0.013, *p* < 0.001) were statistically significant.

Figure 1Estimated means of anxiety and depression of the three latent classes in parallel‐process latent class growth model. *Note: y*‐axis indicates means of anxiety and depression; *x*‐axis indicates the measurement times. A, B, C, and D represents “Means of anxiety and depression”, “Class 1: resistance group”, “Class 2: persistence growth group”, and “Class 3: chronic co‐occurring group”, respectively.

**Figure:**
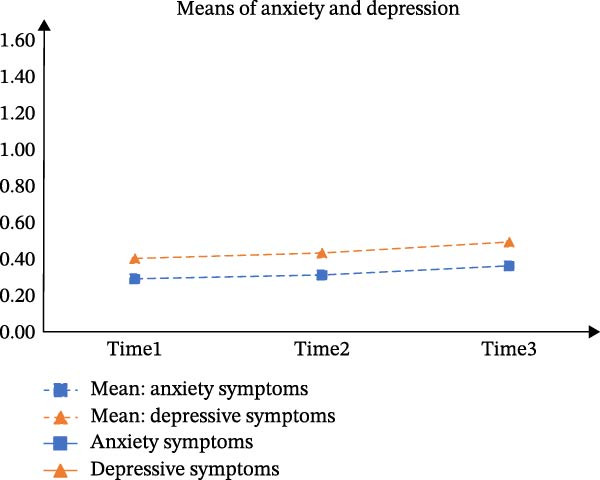
(A)

**Figure:**
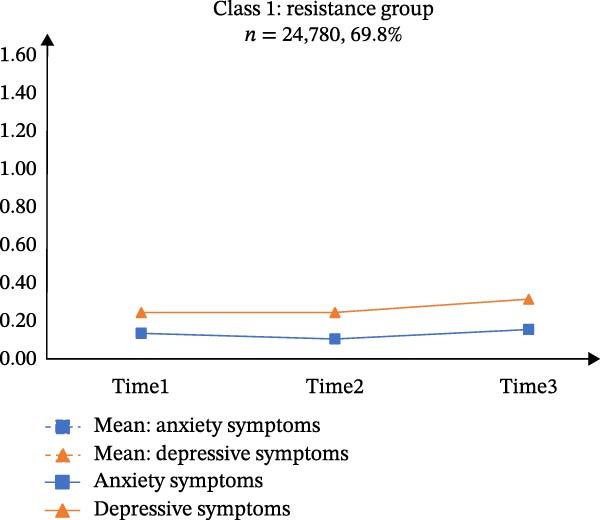
(B)

**Figure:**
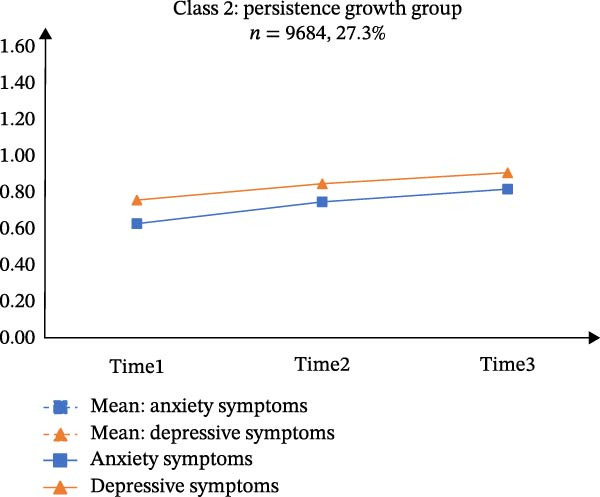
(C)

**Figure:**
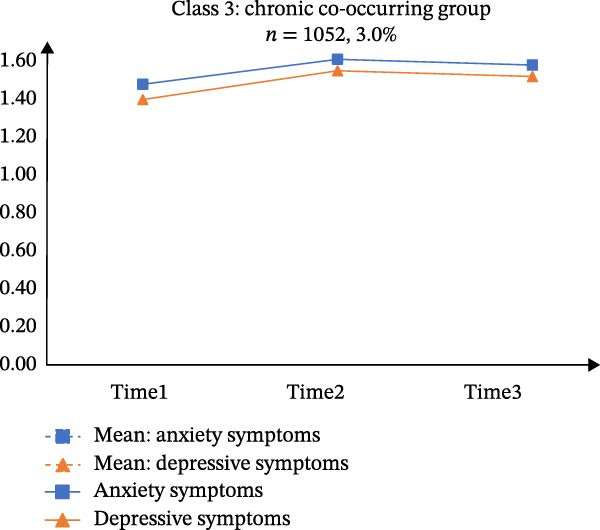
(D)

Table 1 presents the model fit indices for the PP‐LCGM with 1–5 trajectory classes. While both the LMR–LRT and BLRT tests indicated statistical significance for all models, the information criteria showed a declining trend as the number of classes increased. In determining the optimal class solution, the three‐class model had the highest entropy value compared to the four‐ and five‐class models. Based on this criterion, the three‐class solution was selected as the final model.

**Table 1:** Model fit indices for parallel‐process latent class growth models of anxiety and depressive symptoms.

Number of class	AIC	BIC	aBIC	Entropy	LMR–LRT	BLRT	Proportions by classes based on the estimated model
1‐Class	166539.00	166649.21	166607.89	—	—	—	—
2‐Class	128666.68	128819.28	128762.07	0.881	<0.001	<0.001	0.228/0.772
3‐Class	116783.54	116978.53	116905.43	0.897	<0.001	<0.001	0.698/0.272/0.030
4‐Class	107896.02	108133.40	108044.42	0.834	<0.001	<0.001	0.209/0.174/0.025/0.592
5‐Class	99506.64	99786.40	99681.53	0.864	<0.001	<0.001	0.023/0.534/0.150/0.104/0.190

The three‐class solution for anxiety and depressive trajectories is presented in Figure 1B‐D. Class 1 (“*Resistance group*”; *n* = 24,780, 69.8%) exhibited consistently low levels of both anxiety and depressive symptoms across all three waves. Class 2 (“*Persistent growth group*”; *n* = 9,684, 27.3%) was characterized by initially moderate symptoms that increased over time. Class 3 (“*Chronic co-occurring group*”; *n* = 1,052, 3.0%) demonstrated chronically high levels of anxiety and depression across three waves.

Table S4 summarizes the frequency of each symptom from the GAD‐7 (anxiety symptoms) and PHQ‐9 (depressive symptoms) across the three time points. Overall, the prevalence of both anxiety and depressive symptoms increased from T1 to T3 as the pandemic progressed. Among all symptoms, “Anhedonia” exhibited the highest prevalence consistently across all waves (T1, 55.0%; T2, 57.3%; T3, 63.7%).

#### 3.2.1. Network Structures

**Figure 2:**
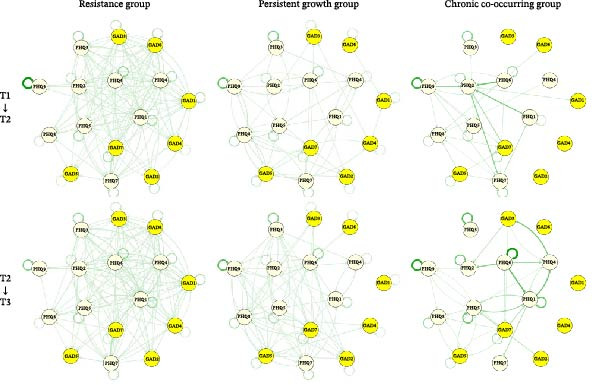
Cross‐lagged panel networks across the heterogeneous co‐developmental trajectories of anxiety and depressive symptoms. A threshold was manually set to OR = 1 to make the figures more interpretable. *Note:* T1, the COVID‐19 outbreak period; T2, the COVID‐19 transitional period; T3, the COVID‐19 control period; COVID‐19, Coronavirus disease 2019. GAD1, nervousness; GAD2, uncontrollable worrying; GAD3, worrying too much; GAD4, trouble relaxing; GAD5, restlessness; GAD6, irritability; GAD7, feeling afraid; PHQ1, anhedonia; PHQ2, depressed mood; PHQ3, sleep problems; PHQ4, lack of energy; PHQ5, appetite; PHQ6, guilt; PHQ7, difficulty concentrating; PHQ8, psychomotor agitation/retardation; PHQ9, suicidal ideation. Abbreviations: GAD, Generalized Anxiety Disorder; PHQ, Patient Health Questionnaire.

Figure 2 and Tables S5–S10 present six CLPN models, depicting the T1→T2 and T2→T3 networks for the resistance, persistent growth, and chronic co‐occurring groups. The maximum possible number of edges in each network is 256. In the resistance group, the T1→T2 network (with 201 estimated edges; network density = 0.785) was less dense than the T2→T3 network (225 edges; network density = 0.879). The number of cross‐lagged edges was also fewer in the T1→T2 network (185) than in the T2→T3 network (210). Across both networks, the strongest cross‐lagged edges consistently linked “Anhedonia (PHQ1)” and “Lack of energy (PHQ4)” (T1→T2: OR = 1.66; T2→T3: OR = 1.89). Within the persistent growth group, both the T1→T2 and T2→T3 networks contained the same number of estimated edges (199; network density = 0.777) and cross‐lagged edges (183). The strongest cross‐lagged edge in the T1→T2 network was from “Restlessness (GAD5)” to “Psychomotor agitation/retardation (PHQ8)” (OR = 1.45), while the reverse direction (OR = 1.47) was the strongest in the T2→T3 network. For the chronic co‐occurring group, network density was greater in the T1→T2 network (66 edges; network density = 0.258) than in the T2→T3 network (50 edges; network density = 0.195). Similarly, more cross‐lagged edges were observed in the T1→T2 network (56) than in the T2→T3 network (41). The edge between “Guilt (PHQ6)” and “Depressed mood (PHQ2)” (OR = 3.75) was the strongest in the T1→T2 network, whereas “Guilt (PHQ6)” to “Anhedonia (PHQ1)” (OR = 4.01) was the strongest in the T2→T3 network.

#### 3.2.2. Network Inference

Table 2 summarizes the standardized in‐EI and out‐EI values across six CLPN networks. In the resistance group, “Anhedonia (PHQ1)” showed the highest out‐EI in both the T1→T2 (1.80) and T2→T3 (1.90) networks. “Lack of energy (PHQ4)” exhibited the highest in‐EI across both timepoints (T1→T2 : 1.79; T2→T3 : 1.75). Within the persistent growth group, “Psychomotor agitation/retardation (PHQ8)” consistently demonstrates the highest out‐EI (T1→T2 : 2.19; T2→T3 : 2.06). For in‐EI, the highest values were observed for “Lack of energy (PHQ4; 2.16)” in the T1→T2 network and “Psychomotor agitation/retardation (PHQ8; 1.95)” in the T2→T3 network. In the chronic co‐occurring group, “Guilt (PHQ6)” had the highest out‐EI in both the T1→T2 (1.85) and T2→T3 (2.78) networks. The highest in‐EI values were found for “Depressed mood (PHQ2; 3.29)” in the T1→T2 network and “Anhedonia (PHQ1; 2.31)” in the T2→T3 network.

**Table 2:** The network inference within six cross‐lagged panel networks across the heterogeneous co‐developmental trajectories of anxiety and depressive symptoms.

Symptoms	T1→T2 network			T2→T3 network		
	Resistance group	Persistence growth group	Chronic co‐occurring group	Resistance group	Persistence growth group	Chronic co‐occurring group
out‐EI						
GAD1	0.33	−1.65	−1.41	0.45	−1.82	−0.59
GAD2	−0.26	−1.22	−1.33	−1.27	0.06	−0.33
GAD3	0.52	−0.73	−0.85	−0.11	−0.65	−0.09
GAD4	−0.62	0.32	−0.88	−0.18	−0.99	−1.05
GAD5	−1.29	**1.46**	−0.05	−1.41	**1.09**	−0.53
GAD6	0.17	−0.51	−0.42	0.28	−1.22	−0.60
GAD7	0.12	0.36	0.70	−0.89	0.79	0.16
PHQ1	**1.80**	0.22	0.96	**1.90**	0.07	**1.64**
PHQ2	0.50	0.12	**1.10**	**1.05**	0.41	−0.53
PHQ3	0.20	0.17	−0.21	0.33	0.55	−0.63
PHQ4	0.97	−1.45	−0.63	0.99	−1.24	0.78
PHQ5	0.32	0.16	−0.48	0.18	−0.12	0.48
PHQ6	0.35	0.64	**1.85**	0.56	0.80	**2.78**
PHQ7	0.53	0.26	0.41	0.69	0.20	−0.65
PHQ8	−1.16	**2.19**	−0.37	−0.84	**2.06**	−0.39
PHQ9	−2.48	−0.34	1.61	−1.73	0.03	−0.44
in‐EI						
GAD1	−0.51	−0.52	−0.16	−0.29	−0.93	−0.75
GAD2	−0.28	0.48	−0.16	0.68	−0.51	−0.75
GAD3	−0.85	−1.00	−0.36	−0.53	−1.04	0.80
GAD4	0.42	−0.12	−0.16	0.06	−0.70	−0.75
GAD5	−1.05	0.34	−0.79	−0.42	0.36	−0.30
GAD6	−0.89	−1.10	−0.72	−0.33	−1.33	−0.75
GAD7	−0.02	0.89	−0.39	−1.05	**1.41**	−0.26
PHQ1	**1.27**	0.17	0.26	0.72	0.48	**2.31**
PHQ2	**1.23**	0.26	**3.29**	**1.42**	0.41	0.96
PHQ3	0.25	−1.51	−0.08	−0.44	−0.28	−0.75
PHQ4	**1.79**	**2.16**	−0.16	**1.75**	0.66	**1.99**
PHQ5	0.28	0.98	0.02	−0.15	**1.27**	0.08
PHQ6	0.02	−1.24	−0.04	−0.22	−1.36	−0.75
PHQ7	0.30	0.35	0.73	0.52	0.10	−0.04
PHQ8	0.34	0.85	0.15	0.76	**1.95**	−0.35
PHQ9	−2.28	−0.98	−1.44	−2.46	−0.48	−0.69

*Note:* T1, the COVID‐19 outbreak period; T2, the COVID‐19 transitional period; T3, the COVID‐19 control period; COVID‐19, Coronavirus disease 2019. GAD1, nervousness; GAD2, uncontrollable worrying; GAD3, worrying too much; GAD4, trouble relaxing; GAD5, restlessness; GAD6, irritability; GAD7, feeling afraid; PHQ1, anhedonia; PHQ2, depressed mood; PHQ3, sleep problems; PHQ4, lack of energy; PHQ5, appetite; PHQ6, guilt; PHQ7, difficulty concentrating; PHQ8, psychomotor agitation/retardation; PHQ9, suicidal ideation. Bold values to identify a manageable number of core symptoms for clinical interpretation, defined a symptom as influential when the out‐EI exceeded 1.

Abbreviations: GAD, Generalized Anxiety Disorder; PHQ, Patient Health Questionnaire.

#### 3.2.3. Network Stability and Accuracy

Bootstrap 95% CIs for all edge weights in CLPN networks, excluding those in the chronic co‐occurring group, were narrow (Figure S3). For instance, mean CI in the persistent growth and resistance groups were 0.97 [0.86, 1.08] and 1.03 [0.92, 1.15] for the T1→T2 network, and 0.98 [0.84, 1.11] and 1.03 [0.92, 1.15] for the T2→T3 network. Bootstrap difference tests for edges and centrality were statistically significant across networks, except for in‐EI centrality in the T1→T2 network of the chronic co‐occurring group (Figures S4–S6). Stability analyses indicated that in‐EI and out‐EI centrality were adequately stable in the resistance (CS coefficients ≥ 0.74) and persistent growth groups (CS coefficients ≥ 0.52; Figure S7). In contrast, both in‐EI and out‐EI stability estimates in the chronic co‐occurring group fell below 0.25 across timepoints (T1→T2 network: in‐EI, 0.07; out‐EI, 0.12; T2→T3 network: in‐EI, 0.00; out‐EI, 0.07). Due to insufficient stability, centrality results for the chronic co‐occurring group are not interpreted in the Discussion section.

#### 3.2.4. Network Replicability

Table 3 presents network similarity comparisons between T1–T2 and T2–T3 across the three trajectory groups. In the resistance group, centrality indices (out‐EI: 0.875, *p* < 0.001; in‐EI: 0.857, *p* < 0.001) and edges replicated (*r* = 0.844, *p*  < 0.001) showed strong and significant correlations between the two temporal networks. Within the persistent growth group, both edge weights (*r*: 0.307–0.979, *p* < 0.001) and centrality indices (out‐EI: 0.841, *p* < 0.001; in‐EI: 0.756, *p* < 0.01) were also significantly correlated between the two networks. In contrast, for the chronic co‐occurring group, neither edge weights nor centrality indices exhibited significant correlations between timepoints, except for edge replication.

**Table 3:** Results of similarities between T1–T2 and T2–T3 networks across the heterogeneous co‐developmental trajectories of anxiety and depressive symptoms.

OR values/variables	Resistance group					Persistence growth group					Chronic co‐occurring group				
	T1→T2		T2→T3			T1→T2		T2→T3			T1→T2		T2→T3		
	M ± SD	*N* (*%*)	M ± SD	*N* (*%*)	*r*	M ± SD	*N* (*%*)	M ± SD	*N* (*%*)		M ± SD	*N* (*%*)	M ± SD	*N* (*%*)	
Edge weights															
All	1.1 ± 0.52	201 (100.0)	1.1 ± 0.39	225 (100.0)	0.001	1.0 ± 0.37	199 (100.0)	1.1 ± 0.41	199 (100.0)	**0.413 ^∗∗∗^**	1.3 ± 0.95	66 (100.0)	1.9 ± 1.35	50 (100.0)	0.093
OR>1	1.3 ± 0.57	139 (69.2)	1.3 ± 0.37	139 (61.8)		1.4 ± 0.53	61 (30.7)	1.3 ± 0.52	84 (42.2)		1.9 ± 0.98	32 (48.5)	2.2 ± 1.37	39 (78.0)	
OR<1	0.8 ± 0.13	62 (30.8)	0.8 ± 0.12	86 (38.2)		0.9 ± 0.07	138 (69.3)	0.9 ± 0.09	115 (57.8)		0.7 ± 0.25	34 (51.5)	0.8 ± 0.20	11 (22.0)	
Autoregressive	2.3 ± 1.23	16(8.0)	2.1 ± 0.61	15 (6.7)	0.114	2.0 ± 0.76	16 (8.0)	2.1 ± 0.85	16 (8.0)	**0.979 ^∗∗∗^**	2.6 ± 1.00	10 (15.2)	3.8 ± 1.53	9 (18.0)	−0.164
OR > 1	2.3 ± 1.23	16 (8.0)	2.2 ± 0.55	14 (6.2)		2.0 ± 0.76	16 (8.0)	2.1 ± 0.85	16 (8.0)		2.6 ± 1.00	10 (15.2)	3.8 ± 1.53	9 (18.0)	
OR < 1			0.95	1 (0.4)											
Cross‐lagged	1.0 ± 0.20	185(92.0)	1.0 ± 0.25	210 (93.3)	0.040	1.0 ± 0.13	183 (92.0)	1.0 ± 0.17	183 (92.0)	**0.307 ^∗∗∗^**	1.0 ± 0.72	56 (84.8)	1.5 ± 0.86	41 (82.0)	0.371
OR > 1	1.2 ± 0.12	123 (61.2)	1.2 ± 0.16	125 (55.6)		1.1 ± 0.11	45 (22.6)	1.2 ± 0.11	68 (34.2)		1.6 ± 0.82	22 (33.3)	1.7 ± 0.88	30 (60.0)	
OR < 1	0.8 ± 0.13	62 (30.8)	0.8 ± 0.12	85 (37.8)		0.9 ± 0.07	138 (69.3)	0.9 ± 0.09	115 (57.8)		0.7 ± 0.25	34 (51.5)	0.8 ± 0.20	11 (22.0)	
Replicated in both network	1.2 ± 0.54	188 (93.5)	1.1 ± 0.42	188 (83.6)	**0.844 ^∗∗∗^**	1.06 ± 0.4	160 (80.4)	1.1 ± 0.45	160 (80.4)	**0.967 ^∗∗∗^**	1.3 ± 0.45	19 (28.8)	1.1 ± 0.32	19 (38.0)	**0.791** **^∗∗∗^**
OR > 1	1.3 ± 0.58	133 (66.2)	1.3 ± 0.39	119 (52.9)		1.4 ± 0.55	58 (29.1)	1.4 ± 0.57	67 (33.7)		2.3 ± 1.14	14 (21.2)	2.8 ± 1.63	17 (34.0)	
OR < 1	0.8 ± 0.13	55 (27.4)	0.8 ± 0.12	69 (30.7)		0.9 ± 0.08	102 (51.3)	0.9 ± 0.09	93 (46.7)		0.7 ± 0.17	5 (7.6)	1.0 ± 0.03	2 (4.0)	
Centrality indices															
In‐EI					**0.857 ^∗∗∗^**					**0.756 ^∗∗^**					0.359
Out‐EI					**0.875 ^∗∗∗^**					**0.841 ^∗∗∗^**					0.492

*Note:* T1, the COVID‐19 outbreak period; T2, the COVID‐19 transitional period; T3, the COVID‐19 control period; COVID‐19, Coronavirus disease 2019. Bold values indicate that the correlation coefficient has statistical significance.

Abbreviations: M, mean; SD, standard deviation.

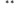
 *p* < 0.01.

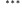
 *p* < 0.001.

## 4. Discussion

This study documents the comorbidity rates of anxiety and depression across the outbreak, initial remission, and control periods of the COVID‐19 pandemic. It also introduces a novel network perspective to examine temporal precedence among their symptoms across the heterogeneous co‐developmental trajectories. Overall, more than 40% of individuals with depression also reported anxiety throughout the different stages of the pandemic. Our findings identified three distinct co‐developmental trajectories of anxiety and depression. More importantly, the symptoms that exerted the strongest influence on other symptoms differed among the networks representing these trajectories. In contrast, the most predictive symptoms within each trajectory network remained consistent over time (T1→T2 vs. T2→T3).

The prevalence of anxiety (T1:11.5%; T2:14.4%; T3:18.3%) and depression (T1:21.7%; T2:26.2%; T3:32.9%) among college students increased gradually as COVID‐19 came under control in China. These trends align with several meta‐analyses that reported a rise in mental health issues over the course of the pandemic [2–4]. Several reasons may explain this escalation. First, the increasing volume and complexity of pandemic‐related information may have adversely affected mental health, particularly among individuals lacking prior social experience [2, 44]. Second, home quarantine and delaying the start of school led to social isolation and online learning fatigue, which likely contributed to psychological distress among students [3, 4, 45]. Third, college students, transitioning from adolescence to adulthood, often struggle to cope with unfamiliar and accumulating stressors [4, 46]. Notably, the comorbidity rate of anxiety and depression among college students in our samples exceeded 40%, which is substantially higher than previously reported rates: 26% among Chinese college students [47] and 32% among Chinese adolescents [48]. Differences in cutoff values of the assessment tool and sample characteristics may partly explain this discrepancy. Two potential mechanisms may underlie the high comorbidity of anxiety and depression. On the one hand, anxiety can foster a sense of helplessness. When such helplessness becomes perceived as insurmountable and pervasive, it may evolve into hopelessness, thereby triggering depressive symptoms [8]. On the another hand, it can lead to persistent activation of the body’s stress response system, particularly the hypothalamic–pituitary–adrenal (HPA) axis. Chronic hyperactivation may deplete key neurotransmitters such as serotonin, norepinephrine, and dopamine, which play critical roles in mood, motivation, and reward processing—functions often impaired in depression [49].

Three distinct joint developmental trajectories of anxiety and depression were identified during the COVID‐19 pandemic: a resistance group (69.8%), a persistent growth group (27.3%), and a chronic co‐occurring group (3.0%). Although few studies have specifically examined the co‐developmental trajectories of anxiety and depression during the pandemic, longitudinal research on their individual symptom trajectories offers partial support for our findings. Previous studies have revealed heterogeneous trajectories of anxiety and depression across different populations, even if the number of identified latent classes varies [11–13]. For instance, Parsons et al. (2022) applied the GMM to identify four anxiety trajectories (consistently low symptoms: 69%; moderate‐decreasing: 9%; near‐severe: 7%; consistently high: 15%) and four depressive symptom trajectories (low‐stable: 60%; moderate‐decreasing: 13%; near‐severe: 17%; high‐stable: 10%). Consistent with our findings, these studies confirm that mental health outcomes follow distinct trajectories during the pandemic. This heterogeneity may reflect not only the uneven societal impact of COVID‐19 but also the heightened vulnerability of specific subgroups to adverse mental health outcomes [11, 50]. Consequently, early intervention, targeted clinical, and social support should be provided to individuals in the persistent growth and chronic co‐occurring groups during the COVID‐19 pandemic.

Among the three identified trajectories, the most influential symptoms—those with the most significant predictive effect on subsequent symptoms—differed across groups. In the chronic co‐occurring group, “Guilt” was the most central symptom; in the persistent growth group, it was “Psychomotor agitation/retardation”; and in the resistance group, “Anhedonia” showed the most substantial predictive influence on other symptoms at follow‐up. Although few studies have examined longitudinal networks of comorbid anxiety and depression, existing cross‐sectional evidence partially supports our findings [20, 51]. For example, previous network analyses have identified “Guilt” or “Psychomotor agitation/retardation” as central symptoms in cross‐sectional networks of anxiety and depression [20, 51]. Guilt is often associated with negative self‐evaluations or self‐concept [52, 53], which may be further distorted in individuals experiencing major mental disorders [54, 55]. According to scar theory, individuals in the persistent growth group who initially reported clinical anxiety and depression may be susceptible to a process in which these early symptoms predict the development of more severe psychopathology over time [26, 55]. Additionally, “Psychomotor agitation/retardation,” a somatic symptom commonly associated with depression [56], has been shown to exert a strong influence within symptom networks of both anxiety and depression [57]. This may help explain its predictive role in the persistent growth group. Taken together, these findings suggest that interventions targeting guilt and psychomotor agitation may help prevent the emergence or reduce the severity of other anxiety and depressive symptoms.

To our knowledge, this is the first large‐scale longitudinal study to examine the comorbidity rates of anxiety and depression during the COVID‐19 pandemic. It also extends existing research by identifying central symptoms within the heterogeneous co‐developmental trajectories of these conditions, highlighting specific symptoms that predict subsequent symptom activation across different pathways. However, several limitations should be noted. First, the use of a college student sample may limit the generalizability of findings to other populations with different age‐related, occupational, or academic stressors. Second, although anxiety and depressive symptoms were assessed using validated self‐report measures (GAD‐7 and PHQ‐9), the absence of structured clinical interviews precludes confirmation of diagnostic status. Third, while the CLPN approach offers insight into potential causal associations between symptoms [15, 16, 18, 25], it cannot establish definitive bidirectional and causal associations between symptoms, nor account for within‐subject variation over time. Fourth, the use of a fixed threshold (out‐EI > 1) to identify bridge symptoms, though consistent with prior work, may limit cross‐study comparability, as absolute expected influence values depend on network scaling and estimation methods. Finally, the estimated network for the chronic co‐occurring group exhibited low stability and accuracy, limiting precise analysis of symptom interactions in this subgroup. This may be attributed to several reasons: (1) a smaller sample size in the chronic co‐occurring group compared to other subgroups; (2) limited covariance due to uniformly high (or low) symptom scores among most participants in this group; and (3) greater heterogeneity in underlying illness trajectories or phenotypic presentations within the chronic co‐occurring group relative to the non‐chronic group.

## 5. Conclusions

Our study revealed a high comorbidity rate (≥40%) between anxiety and depression during the pandemic, identified distinct co‐development trajectories of these conditions, and uncovered central and predictive symptoms within each trajectory. Network analysis highlighted “Guilt” as the most influential symptom in the chronic co‐occurring group and “Psychomotor agitation/retardation” in the persistent growth group. These findings emphasize the importance of tailored and individualized interventions targeting specific subpopulations during the pandemic, which may help prevent the worsening of symptoms and reduce the overall symptom burden over time.

## Author Contributions

All authors were involved in conceptualizing the study. Zijuan Ma performed the data preprocessing and analysis with technical input from Zhijun Yu and Shaochen Zhao. Clinical expertise was provided by Dongfang Wang, Yang Li, and Fang Fan. Zijuan Ma drafted the manuscript, and all other authors provided critical feedback. Xiao‐Yan Chen revised the manuscript. Dongfang Wang and Fang Fan supervised this work.

## Funding

This work was supported by the National Natural Science Foundation of China (Grants 31871129 and 32271135) and the 2023 Young Teacher Research and Training Fund Project of South China Normal University: Humanities and Social Sciences (Grant 23SK16).

## Disclosure

All authors approved the final version prior to submission.

## Conflicts of Interest

The authors declare no conflicts of interest.

## Supporting Information

Additional supporting information can be found online in the Supporting Information section.

## Supporting information


**Supporting Information** The optimal number of trajectory memberships was determined based on several criteria (Nylund‐Gibson et al.) [14]: (1) lower information fit indices, including Akaike Information Criteria (AIC), Bayesian Information Criterion (BIC), and sample size adjusted BIC (ABIC); (2) statistically significant *p* ‐values (<0.001) for both the Lo‐Mendell‐Rubin likelihood ratio test (LMR‐LRT) and bootstrap likelihood ratio tests (BLRT); (3) higher entropy values (greater than 0.80); (4) sufficient sample sizes in each class. Moreover, the theoretical and practical significance of clinical research was also considered (Nagin and Odgers) [37]. Table S1. Demographic and pandemic‐related factors (*N* = 35 516). Note: COVID‐19, Coronavirus disease 2019. Table S2. Mean, standard deviations (SD), and correlations for the total scores of anxiety and depression. Table S3. Longitudinal measurement invariance for anxiety and depressive symptoms across three measurements. Table S4. Basic information and the frequency of each symptom in the GAD‐7 (anxiety symptoms) and PHQ‐9 (depressive symptoms; *N* = 35 516). Note: YSIS, Youth Self Rating Insomnia Scale; GAD‐7, Generalized Anxiety Disorder‐7; PHQ‐9, Patient Health Questionnaire‐9; T1, the COVID‐19 outbreak period; T2, the COVID‐19 transitional period; T3, the COVID‐19 control period; COVID‐19, Coronavirus disease 2019. Table S5. Weighted adjacency matrix of resistance group within the T1→T2 network. Note: GAD, Generalized Anxiety Disorder; PHQ, Patient Health Questionnaire; T1, the COVID‐19 outbreak period; T2, the COVID‐19 transitional period; COVID‐19, Coronavirus disease 2019. Table S6. Weighted adjacency matrix of persistence growth group within the T1→T2 network. Note: GAD, Generalized Anxiety Disorder; PHQ, Patient Health Questionnaire; T1, the COVID‐19 outbreak period; T2, the COVID‐19 transitional period; COVID‐19, Coronavirus disease 2019. Table S7. Weighted adjacency matrix of chronic co‐occurring group within the T1→T2 network. Note: GAD, Generalized Anxiety Disorder; PHQ, Patient Health Questionnaire; T1, the COVID‐19 outbreak period; T2, the COVID‐19 transitional period; COVID‐19, Coronavirus disease 2019. Table S8. Weighted adjacency matrix of resistance group within the T2→T3 network. Note: GAD, Generalized Anxiety Disorder; PHQ, Patient Health Questionnaire; T2, the COVID‐19 transitional period; T3, the COVID‐19 control period; COVID‐19, Coronavirus disease 2019. Table S9. Weighted adjacency matrix of persistence growth group within the T2→T3 network. Note: GAD, Generalized Anxiety Disorder; PHQ, Patient Health Questionnaire; T2, the COVID‐19 transitional period; T3, the COVID‐19 control period; COVID‐19, Coronavirus disease 2019. Table S10. Weighted adjacency matrix of chronic co‐occurring group within the T2→T3 network. Note: GAD, Generalized Anxiety Disorder; PHQ, Patient Health Questionnaire; T2, the COVID‐19 transitional period; T3, the COVID‐19 control period; COVID‐19, Coronavirus disease 2019. Figure S1. The national pandemic trend of the 2019 coronavirus disease (COVID‐19) in China and sampling times. This figure is reproduced from previous studies [13, 29]. Figure S2. Geographical distribution of participating colleges and universities. This figure is reproduced from a previous study [58]. Figure S3. Bootstrapped 95% confidence intervals around each edge weight across the heterogeneous co‐developmental trajectories of anxiety and depressive symptoms. Note: The gray area represents the 95% Confidence Intervals of edge weights. Red lines indicate the edge weight in the estimated sample network, while black lines indicate the values of each edge weight, ordered from the highest to the lowest value. T1, the COVID‐19 outbreak period; T2, the COVID‐19 transitional period; T3, the COVID‐19 control period; COVID‐19, Coronavirus disease 2019. Figure S4. Edge weight difference tests across the heterogeneous co‐developmental trajectories of anxiety and depressive symptoms. Note: Gray boxes indicate edges that do not significantly differ from one‐another. Black boxes represent edges with significant difference from one‐another (α = 0.05). Blue boxes in the edge‐weight plot indicate positive correlations, and orange boxes in the edge‐weight plot indicate negative correlations. T1, the COVID‐19 outbreak period; T2, the COVID‐19 transitional period; T3, the COVID‐19 control period; COVID‐19, Coronavirus disease 2019. Figure S5. Centrality difference tests of Out Expected Influence across the heterogeneous co‐developmental trajectories of anxiety and depressive symptoms. Note: Black boxes indicate symptoms that significantly differ in centrality (*p* < 0.05), and gray boxes indicate symptoms whose centrality does not significantly differ. T1, the COVID‐19 outbreak period; T2, the COVID‐19 transitional period; T3, the COVID‐19 control period; COVID‐19, Coronavirus disease 2019. Figure S6. Centrality difference tests of In Expected Influence across the heterogeneous co‐developmental trajectories of anxiety and depressive symptoms. Note: Black boxes indicate symptoms that significantly differ in centrality (*p* < 0.05), and gray boxes indicate symptoms whose centrality does not significantly differ. T1, the COVID‐19 outbreak period; T2, the COVID‐19 transitional period; T3, the COVID‐19 control period; COVID‐19, Coronavirus disease 2019. Figure S7. Stability of centrality measures across the heterogeneous co‐developmental trajectories of anxiety and depressive symptoms. Note: The y‐axis represents the average correlations between the original network’s centrality indices and the centrality indices from the networks that were re‐estimated after excluding increasing percentages of cases. T1, the COVID‐19 outbreak period; T2, the COVID‐19 transitional period; T3, the COVID‐19 control period; COVID‐19, Coronavirus disease 2019.

## Data Availability

The data that support the findings of this study are available upon request from the corresponding author. The data are not publicly available due to privacy or ethical restrictions.
