# Supplementary material for: Disentangling Heterogeneity in the Co‐Developmental of Anxiety and Depression During the COVID‐19 Pandemic: Insights From Cross‐Lagged Panel Network
Source: Depress Anxiety. 2026 Feb 23;2026:6008342. doi: 10.1155/da/6008342 (PMC12929178; doi:10.1155/da/6008342)
Supplement: Supplementary file 1 — Supporting Information The optimal number of trajectory memberships was determined based on several criteria (Nylund‐Gibson et al.) [14]: (1) lower information fit indices, including Akaike Information Criteria (AIC), Bayesian Information Criterion (BIC), and sample size adjusted BIC (ABIC); (2) statistically significant p ‐values (<0.001) for both the Lo‐Mendell‐Rubin likelihood ratio test (LMR‐LRT) and bootstrap likelihood ratio tests (BLRT); (3) higher entropy values (greater than 0.80); (4) sufficient sample sizes in each class. Moreover, the theoretical and practical significance of clinical research was also considered (Nagin and Odgers) [37]. Table S1. Demographic and pandemic‐related factors (N = 35 516). Note: COVID‐19, Coronavirus disease 2019. Table S2. Mean, standard deviations (SD), and correlations for the total scores of anxiety and depression. Table S3. Longitudinal measurement invariance for anxiety and depressive symptoms across three measurements. Table S4. Basic information and the frequency of each symptom in the GAD‐7 (anxiety symptoms) and PHQ‐9 (depressive symptoms; N = 35 516). Note: YSIS, Youth Self Rating Insomnia Scale; GAD‐7, Generalized Anxiety Disorder‐7; PHQ‐9, Patient Health Questionnaire‐9; T1, the COVID‐19 outbreak period; T2, the COVID‐19 transitional period; T3, the COVID‐19 control period; COVID‐19, Coronavirus disease 2019. Table S5. Weighted adjacency matrix of resistance group within the T1→T2 network. Note: GAD, Generalized Anxiety Disorder; PHQ, Patient Health Questionnaire; T1, the COVID‐19 outbreak period; T2, the COVID‐19 transitional period; COVID‐19, Coronavirus disease 2019. Table S6. Weighted adjacency matrix of persistence growth group within the T1→T2 network. Note: GAD, Generalized Anxiety Disorder; PHQ, Patient Health Questionnaire; T1, the COVID‐19 outbreak period; T2, the COVID‐19 transitional period; COVID‐19, Coronavirus disease 2019. Table S7. Weighted adjacency matrix of chronic co‐occurring group within th [file DA-2026-6008342-s001.docx]

**Supplementary Table 1.** Demographic and pandemic-related factors (*N* = 35 516).

| **Variable** | |  | ***n*** | **%** |
| --- | --- | --- | --- | --- |
| **Sex** | |  |  |  |
|  | Male |  | 9244 | 26.0 |
|  | Female |  | 26272 | 74.0 |
| **Age** | |  |  |  |
|  | Below 23 years |  | 31933 | 89.9 |
|  | Equal to or above 23 years |  | 3583 | 10.1 |
| **Provincial-level epidemic severity** | |  |  |  |
|  | Mild to moderate |  | 35246 | 99.2 |
|  | Severe |  | 270 | 0.8 |
| **Infected cases in the community or village** | |  |  |  |
|  | No |  | 2352 | 6.6 |
|  | Yes |  | 33164 | 93.4 |
| **Relatives or acquaintances being infected with COVID-19** | |  |  |  |
|  | Nobody or don't know |  | 35116 | 98.9 |
|  | Confirmed or suspected |  | 400 | 1.1 |
| **Exposure to media coverage of the COVID-19** | |  |  |  |
|  | < 3 hours/day |  | 29924 | 84.3 |
|  | ≥3 hours/day |  | 5592 | 15.7 |

***Note:*** COVID-19, Coronavirus disease 2019.

**Supplementary Table 2.** Mean, standard deviations (SD), and correlations for the total scores of anxiety and depression.

|  | M ± SD | 1 | 2 | 3 | 4 | 5 | 6 |
| --- | --- | --- | --- | --- | --- | --- | --- |
| 1.Anxiety_T1 | 2.12 ± 3.03 | 1 |  |  |  |  |  |
| 2.Anxiety_T2 | 2.22 ± 3.16 | .55*** | 1 |  |  |  |  |
| 3.Anxiety_T3 | 2.62 ± 3.41 | .50*** | .59*** | 1 |  |  |  |
| 4.Depression_T1 | 3.72 ± 3.82 | .74*** | .53*** | .50*** | 1 |  |  |
| 5.Depression_T2 | 3.97 ± 3.98 | .52*** | .78*** | .58*** | .61*** | 1 |  |
| 6.Depression_T3 | 4.53 ± 4.15 | .48*** | .56*** | .80*** | .56*** | .65*** | 1 |

**Supplementary Table 3.** Longitudinal measurement invariance for anxiety and depressive symptoms across three measurements.

| **Mode** | | | ***χ*^2^** | ***df*** | **CFI** | **TLI** | **RMSEA** | **90%CI** | **△CFI** |
| --- | --- | --- | --- | --- | --- | --- | --- | --- | --- |
|  | **Anxiety symptoms** | |  |  |  |  |  |  |  |
|  |  | Configural invariance | 7397.43*** | 165 | 0.976 | 0.969 | 0.035 | [0.034,0.036] |  |
|  |  | Metric invariance | 8089.21*** | 177 | 0.973 | 0.968 | 0.035 | [0.035,0.036] | 0.003 |
|  |  | Scalar invariance | 11728.92*** | 192 | 0.966 | 0.962 | 0.041 | [0.041,0.042] | 0.007 |
|  | **Depressive symptoms** | |  |  |  |  |  |  |  |
|  |  | Configural invariance | 17598.92*** | 294 | 0.947 | 0.937 | 0.041 | [0.040,0.041] |  |
|  |  | Metric invariance | 18293.82*** | 310 | 0.945 | 0.938 | 0.04 | [0.040,0.041] | 0.002 |
|  |  | Scalar invariance | 22179.76*** | 331 | 0.938 | 0.934 | 0.043 | [0.043,0.044] | 0.007 |

**Supplementary Table 4.** Basic information and the frequency of each symptom in the GAD-7 (anxiety symptoms), and PHQ-9 (depressive symptoms; *N*=35 516).

| **Item** | | | **Short name** | **Label** | **T1** | | **T2** | | **T3** | |
| --- | --- | --- | --- | --- | --- | --- | --- | --- | --- | --- |
|  |  |  |  |  | **N** | **%** | **N** | **%** | **N** | **%** |
|  | **Anxiety symptoms** | |  |  |  |  |  |  |  |  |
|  |  | Feel nervous, anxious, or on edge | Nervousness | GAD1 | 11679 | 32.9 | 13029 | 36.7 | 15089 | 42.5 |
|  |  | Not being able to stop or control worrying | Uncontrollable worrying | GAD2 | 9297 | 26.2 | 9040 | 25.5 | 10831 | 30.5 |
|  |  | Worrying too much about different things | Worry too much | GAD3 | 11432 | 32.2 | 10967 | 30.9 | 12757 | 35.9 |
|  |  | Trouble relaxing | Trouble relaxing | GAD4 | 8610 | 24.2 | 10366 | 29.2 | 12605 | 35.5 |
|  |  | Being so restless that it's hard to sit still | Restlessness | GAD5 | 5823 | 16.4 | 7260 | 20.4 | 9024 | 25.4 |
|  |  | Becoming easily annoyed or irritable | Irritability | GAD6 | 10583 | 29.8 | 12129 | 34.2 | 14294 | 40.2 |
|  |  | Feeling afraid as if something awful might happen | Feeling afraid | GAD7 | 9179 | 25.8 | 7342 | 20.7 | 7778 | 21.9 |
|  | **Depressive symptoms** | |  |  |  |  |  |  |  |  |
|  |  | Little interest or pleasure in doing things | Anhedonia | PHQ1 | 19545 | 55.0 | 20368 | 57.3 | 22629 | 63.7 |
|  |  | Feeling down, depressed or hopeless | Depressed mood | PHQ2 | 15074 | 42.4 | 16761 | 47.2 | 19429 | 54.7 |
|  |  | Trouble falling or staying asleep or sleeping too much | Sleep problem | PHQ3 | 15462 | 43.5 | 16381 | 46.1 | 17694 | 49.8 |
|  |  | Feeling tired or having little energy | Lack of energy | PHQ4 | 16714 | 47.1 | 18667 | 52.6 | 21067 | 59.3 |
|  |  | Poor appetite or overeating | Appetite | PHQ5 | 13412 | 37.8 | 13518 | 38.1 | 16666 | 46.9 |
|  |  | Feeling bad about yourself | Guilt | PHQ6 | 11313 | 31.9 | 13203 | 37.2 | 15119 | 42.6 |
|  |  | Trouble concentrating on things | Difficulty concentration | PHQ7 | 12039 | 33.9 | 13607 | 38.3 | 15356 | 43.2 |
|  |  | Moving or speaking so slowly that other people could have noticed | Psychomotor agitation/retardation | PHQ8 | 5729 | 16.1 | 7561 | 21.3 | 9591 | 27.0 |
|  |  | Thoughts that you would be better off dead or of hurting yourself in some way | Suicidal ideation | PHQ9 | 2580 | 7.3 | 3356 | 9.4 | 4392 | 12.4 |

***Note:*** YSIS, Youth Self Rating Insomnia Scale; GAD-7, Generalized Anxiety Disorder-7; PHQ-9, Patient Health Questionnaire-9; T1, the COVID-19 outbreak period; T2, the COVID-19 transitional period; T3, the COVID-19 control period; COVID-19, Coronavirus disease 2019.

**Supplementary Table 5**. Weighted adjacency matrix of resistance group within T1→T2 network.

|  | GAD1 | GAD2 | GAD3 | GAD4 | GAD5 | GAD6 | GAD7 | PHQ1 | PHQ2 | PHQ3 | PHQ4 | PHQ5 | PHQ6 | PHQ7 | PHQ8 | PHQ9 |
| --- | --- | --- | --- | --- | --- | --- | --- | --- | --- | --- | --- | --- | --- | --- | --- | --- |
| GAD1 | 1.00 | 1.00 | 1.00 | 1.00 | 0.70 | 0.61 | 0.47 | 0.63 | 1.34 | 1.00 | 1.00 | 1.00 | 1.00 | 0.42 | 1.00 | 0.75 |
| GAD2 | 1.00 | 1.00 | 1.00 | 1.00 | 0.82 | 1.00 | 1.00 | 1.00 | 0.12 | 1.00 | 1.00 | 1.00 | 1.00 | 0.54 | 1.00 | 0.57 |
| GAD3 | 1.00 | 1.00 | 1.00 | 1.00 | 0.90 | 1.00 | 1.00 | 0.93 | 0.50 | 1.00 | 1.00 | 1.00 | 1.00 | 0.73 | 1.00 | 0.75 |
| GAD4 | 1.00 | 1.00 | 1.00 | 1.00 | 1.00 | 1.00 | 0.87 | 1.00 | 0.64 | 1.00 | 1.00 | 1.00 | 1.00 | 1.00 | 0.39 | 0.87 |
| GAD5 | 1.00 | 1.00 | 1.00 | 1.00 | 1.82 | 1.00 | 1.11 | 1.00 | 0.31 | 1.00 | 1.00 | 1.00 | 1.00 | 1.17 | 1.48 | 1.00 |
| GAD6 | 1.00 | 1.00 | 1.00 | 1.00 | 1.00 | 1.00 | 1.00 | 1.00 | 0.17 | 1.00 | 1.00 | 1.00 | 1.00 | 1.00 | 1.32 | 1.00 |
| GAD7 | 1.00 | 1.00 | 1.00 | 1.00 | 1.00 | 1.00 | 2.36 | 1.00 | 2.27 | 1.00 | 1.00 | 1.00 | 1.00 | 1.00 | 1.00 | 1.00 |
| PHQ1 | 1.00 | 1.00 | 1.00 | 1.00 | 1.00 | 1.00 | 1.00 | 1.68 | 3.23 | 1.00 | 1.00 | 1.00 | 1.00 | 0.63 | 1.00 | 0.83 |
| PHQ2 | 1.00 | 1.00 | 1.00 | 1.00 | 1.00 | 1.00 | 1.00 | 1.00 | 2.25 | 1.00 | 1.00 | 1.00 | 1.00 | 2.90 | 1.00 | 1.00 |
| PHQ3 | 1.00 | 1.00 | 1.00 | 1.00 | 1.00 | 1.00 | 1.00 | 1.00 | 1.00 | 3.06 | 1.00 | 1.00 | 1.00 | 1.00 | 1.00 | 0.83 |
| PHQ4 | 1.00 | 1.00 | 1.00 | 1.00 | 1.00 | 0.98 | 1.00 | 1.00 | 0.14 | 1.00 | 1.00 | 1.00 | 1.00 | 1.00 | 1.03 | 1.00 |
| PHQ5 | 1.00 | 1.00 | 0.80 | 1.00 | 0.95 | 0.85 | 1.00 | 1.00 | 0.73 | 1.08 | 1.00 | 2.31 | 1.00 | 1.00 | 1.00 | 1.00 |
| PHQ6 | 1.00 | 1.00 | 1.00 | 1.00 | 1.00 | 1.00 | 1.00 | 1.00 | 3.75 | 1.00 | 1.00 | 1.00 | 2.93 | 1.15 | 1.00 | 1.19 |
| PHQ7 | 1.00 | 1.00 | 1.00 | 1.00 | 1.00 | 1.00 | 1.00 | 1.87 | 1.00 | 1.00 | 1.00 | 1.00 | 1.00 | 2.53 | 1.01 | 0.94 |
| PHQ8 | 1.00 | 1.00 | 1.00 | 1.00 | 1.00 | 1.00 | 1.16 | 1.00 | 0.41 | 1.00 | 1.00 | 1.00 | 1.00 | 1.00 | 2.02 | 1.00 |
| PHQ9 | 1.00 | 1.00 | 1.00 | 1.00 | 1.00 | 0.99 | 1.16 | 1.00 | 2.86 | 1.00 | 1.00 | 1.17 | 1.11 | 1.35 | 1.08 | 5.19 |

***Note:*** GAD, Generalized Anxiety Disorder; PHQ, Patient Health Questionnaire; T1, the COVID-19 outbreak period; T2, the COVID-19 transitional period; COVID-19, Coronavirus disease 2019.

**Supplementary Table 6.** Weighted adjacency matrix of persistence growth group within T1→T2 network.

|  | GAD1 | GAD2 | GAD3 | GAD4 | GAD5 | GAD6 | GAD7 | PHQ1 | PHQ2 | PHQ3 | PHQ4 | PHQ5 | PHQ6 | PHQ7 | PHQ8 | PHQ9 |
| --- | --- | --- | --- | --- | --- | --- | --- | --- | --- | --- | --- | --- | --- | --- | --- | --- |
| GAD1 | 1.38 | 0.93 | 0.91 | 0.93 | 0.79 | 0.97 | 0.86 | 1.00 | 0.99 | 0.93 | 0.90 | 0.77 | 0.79 | 0.81 | 0.67 | 0.71 |
| GAD2 | 0.87 | 1.25 | 1.00 | 0.94 | 1.00 | 0.84 | 1.00 | 0.82 | 0.87 | 0.78 | 0.83 | 0.85 | 0.87 | 0.84 | 0.93 | 0.91 |
| GAD3 | 0.98 | 1.00 | 1.45 | 1.00 | 0.82 | 1.00 | 1.00 | 0.95 | 1.00 | 0.89 | 0.94 | 0.93 | 1.00 | 0.81 | 0.84 | 0.69 |
| GAD4 | 1.02 | 1.00 | 1.06 | 1.28 | 1.05 | 0.96 | 1.00 | 1.00 | 0.91 | 0.95 | 1.00 | 0.97 | 0.97 | 0.97 | 0.99 | 1.00 |
| GAD5 | 1.00 | 1.07 | 1.00 | 1.16 | 1.77 | 0.99 | 1.18 | 0.87 | 1.00 | 0.97 | 0.83 | 1.10 | 0.98 | 1.11 | 1.45 | 1.23 |
| GAD6 | 1.05 | 0.85 | 0.89 | 1.00 | 0.97 | 1.40 | 0.80 | 0.95 | 1.00 | 1.00 | 0.93 | 1.00 | 0.99 | 0.80 | 0.94 | 0.88 |
| GAD7 | 1.00 | 1.15 | 1.05 | 0.94 | 0.98 | 0.96 | 1.75 | 1.00 | 0.93 | 0.97 | 0.89 | 0.94 | 1.00 | 1.00 | 1.08 | 1.00 |
| PHQ1 | 1.00 | 0.83 | 0.98 | 1.00 | 0.93 | 1.00 | 0.85 | 1.85 | 1.00 | 0.91 | 1.21 | 1.13 | 1.00 | 1.23 | 0.97 | 0.69 |
| PHQ2 | 1.00 | 0.98 | 0.95 | 0.93 | 0.94 | 0.93 | 1.00 | 1.00 | 1.73 | 0.87 | 1.13 | 0.84 | 1.01 | 0.96 | 1.00 | 1.13 |
| PHQ3 | 0.93 | 0.94 | 0.87 | 0.97 | 0.99 | 1.00 | 1.00 | 1.00 | 0.94 | 3.25 | 1.30 | 1.13 | 0.84 | 0.94 | 0.85 | 1.01 |
| PHQ4 | 0.90 | 0.79 | 0.85 | 0.82 | 0.86 | 0.90 | 0.79 | 1.16 | 0.93 | 0.92 | 1.29 | 0.91 | 0.74 | 0.99 | 0.83 | 0.76 |
| PHQ5 | 0.86 | 0.90 | 0.86 | 0.95 | 0.94 | 1.00 | 0.97 | 1.00 | 1.00 | 1.08 | 1.26 | 2.16 | 0.90 | 0.98 | 1.02 | 0.98 |
| PHQ6 | 0.95 | 1.06 | 1.00 | 1.00 | 0.98 | 1.00 | 1.00 | 1.00 | 1.11 | 1.00 | 1.00 | 0.96 | 2.49 | 1.05 | 1.00 | 1.06 |
| PHQ7 | 1.00 | 0.96 | 0.97 | 0.96 | 1.00 | 0.91 | 0.94 | 1.07 | 1.00 | 1.00 | 1.09 | 0.95 | 1.00 | 2.07 | 1.08 | 0.86 |
| PHQ8 | 1.00 | 1.16 | 1.02 | 1.00 | 1.36 | 0.97 | 1.28 | 1.00 | 0.92 | 1.00 | 0.89 | 1.34 | 1.00 | 1.24 | 2.25 | 1.45 |
| PHQ9 | 0.89 | 1.00 | 0.92 | 0.90 | 1.00 | 0.91 | 1.04 | 0.75 | 1.00 | 0.98 | 0.76 | 0.91 | 1.21 | 0.88 | 1.07 | 3.98 |

***Note:*** GAD, Generalized Anxiety Disorder; PHQ, Patient Health Questionnaire; T1, the COVID-19 outbreak period; T2, the COVID-19 transitional period; COVID-19, Coronavirus disease 2019.

**Supplementary Table 7.** Weighted adjacency matrix of chronic co-occurring group within T1→T2 network.

|  | GAD1 | GAD2 | GAD3 | GAD4 | GAD5 | GAD6 | GAD7 | PHQ1 | PHQ2 | PHQ3 | PHQ4 | PHQ5 | PHQ6 | PHQ7 | PHQ8 | PHQ9 |
| --- | --- | --- | --- | --- | --- | --- | --- | --- | --- | --- | --- | --- | --- | --- | --- | --- |
| GAD1 | 2.18 | 1.19 | 1.25 | 1.25 | 1.00 | 1.24 | 1.00 | 1.12 | 1.17 | 1.10 | 1.19 | 1.00 | 1.05 | 1.02 | 0.88 | 0.70 |
| GAD2 | 1.01 | 1.80 | 1.06 | 1.00 | 1.00 | 1.00 | 1.12 | 0.97 | 0.94 | 0.93 | 0.96 | 0.93 | 1.00 | 1.00 | 1.00 | 1.00 |
| GAD3 | 1.13 | 1.18 | 1.93 | 1.22 | 1.00 | 1.14 | 1.31 | 1.13 | 1.09 | 1.08 | 1.04 | 1.00 | 1.17 | 1.05 | 1.01 | 1.00 |
| GAD4 | 1.00 | 1.00 | 1.00 | 1.23 | 1.00 | 0.82 | 1.00 | 0.92 | 1.00 | 0.91 | 0.88 | 0.88 | 0.89 | 1.00 | 0.99 | 0.88 |
| GAD5 | 0.80 | 0.88 | 0.67 | 0.97 | 1.21 | 0.81 | 0.90 | 0.77 | 0.84 | 0.76 | 0.75 | 0.79 | 0.87 | 0.97 | 1.00 | 0.99 |
| GAD6 | 1.06 | 1.00 | 1.02 | 1.09 | 1.06 | 2.07 | 1.00 | 1.15 | 1.13 | 1.14 | 1.25 | 1.08 | 1.07 | 1.00 | 1.07 | 0.70 |
| GAD7 | 1.04 | 1.18 | 1.24 | 1.00 | 1.00 | 1.03 | 2.42 | 1.10 | 1.00 | 1.11 | 1.10 | 1.07 | 1.00 | 1.04 | 1.00 | 0.83 |
| PHQ1 | 1.31 | 1.24 | 1.23 | 1.23 | 1.00 | 1.26 | 1.05 | 2.45 | 1.38 | 1.35 | 1.66 | 1.31 | 1.24 | 1.54 | 1.27 | 1.14 |
| PHQ2 | 1.10 | 1.00 | 1.13 | 1.17 | 1.02 | 1.07 | 1.00 | 1.06 | 1.94 | 1.09 | 1.12 | 1.08 | 1.19 | 1.01 | 1.21 | 1.25 |
| PHQ3 | 1.04 | 1.00 | 1.00 | 1.13 | 1.00 | 1.08 | 1.00 | 1.19 | 1.09 | 2.58 | 1.32 | 1.28 | 0.99 | 1.02 | 1.00 | 0.76 |
| PHQ4 | 1.25 | 1.00 | 1.02 | 1.11 | 1.07 | 1.21 | 1.00 | 1.53 | 1.24 | 1.29 | 2.05 | 1.39 | 1.11 | 1.20 | 1.09 | 1.00 |
| PHQ5 | 1.00 | 1.00 | 1.00 | 1.00 | 1.00 | 1.08 | 1.00 | 1.22 | 1.09 | 1.27 | 1.29 | 2.20 | 1.00 | 1.18 | 1.10 | 0.91 |
| PHQ6 | 1.08 | 1.00 | 1.06 | 1.00 | 1.06 | 1.03 | 1.08 | 1.03 | 1.26 | 0.99 | 1.10 | 1.00 | 2.98 | 1.00 | 1.14 | 1.37 |
| PHQ7 | 1.07 | 1.00 | 1.05 | 1.12 | 1.18 | 1.05 | 1.00 | 1.36 | 1.13 | 1.03 | 1.16 | 1.04 | 1.18 | 2.17 | 1.20 | 1.00 |
| PHQ8 | 0.75 | 1.00 | 0.80 | 0.81 | 1.01 | 0.75 | 1.00 | 0.82 | 0.85 | 0.79 | 0.79 | 0.99 | 0.73 | 0.95 | 1.59 | 1.00 |
| PHQ9 | 0.64 | 0.68 | 0.59 | 0.54 | 0.64 | 0.52 | 1.00 | 0.63 | 0.73 | 0.72 | 0.61 | 0.73 | 0.99 | 0.62 | 0.64 | 6.61 |

***Note:*** GAD, Generalized Anxiety Disorder; PHQ, Patient Health Questionnaire; T1, the COVID-19 outbreak period; T2, the COVID-19 transitional period; COVID-19, Coronavirus disease 2019.

**Supplementary Table 8.** Weighted adjacency matrix of resistance group within T2→T3 network.

|  | GAD1 | GAD2 | GAD3 | GAD4 | GAD5 | GAD6 | GAD7 | PHQ1 | PHQ2 | PHQ3 | PHQ4 | PHQ5 | PHQ6 | PHQ7 | PHQ8 | PHQ9 |
| --- | --- | --- | --- | --- | --- | --- | --- | --- | --- | --- | --- | --- | --- | --- | --- | --- |
| GAD1 | 1.00 | 1.00 | 1.00 | 1.00 | 0.99 | 1.00 | 1.00 | 1.00 | 1.00 | 1.00 | 0.92 | 1.00 | 1.00 | 1.00 | 1.00 | 1.00 |
| GAD2 | 1.00 | 1.00 | 1.00 | 1.00 | 1.00 | 1.00 | 1.00 | 0.68 | 1.00 | 1.00 | 1.00 | 1.84 | 1.00 | 1.00 | 1.00 | 0.94 |
| GAD3 | 1.00 | 1.00 | 1.00 | 1.00 | 1.00 | 1.00 | 1.03 | 1.00 | 1.83 | 1.00 | 1.12 | 1.00 | 1.00 | 1.00 | 1.00 | 1.00 |
| GAD4 | 1.00 | 1.00 | 1.00 | 1.00 | 1.00 | 1.00 | 1.00 | 0.37 | 1.00 | 1.00 | 1.00 | 0.53 | 1.00 | 1.00 | 1.00 | 1.00 |
| GAD5 | 1.00 | 1.00 | 1.00 | 1.00 | 1.80 | 1.00 | 1.28 | 0.62 | 1.00 | 1.00 | 1.00 | 1.04 | 1.00 | 1.00 | 1.10 | 1.00 |
| GAD6 | 1.00 | 1.00 | 1.00 | 1.00 | 1.00 | 1.00 | 1.00 | 1.00 | 1.00 | 1.00 | 0.87 | 1.00 | 1.00 | 1.00 | 1.00 | 1.00 |
| GAD7 | 1.00 | 1.00 | 1.00 | 1.00 | 1.03 | 1.00 | 2.30 | 2.12 | 1.00 | 1.00 | 1.00 | 1.08 | 1.00 | 1.00 | 1.23 | 1.07 |
| PHQ1 | 1.00 | 1.00 | 1.00 | 1.00 | 1.00 | 1.00 | 1.00 | 3.85 | 1.00 | 1.00 | 3.73 | 1.91 | 1.00 | 2.11 | 1.00 | 1.00 |
| PHQ2 | 1.00 | 1.00 | 1.00 | 1.00 | 1.00 | 1.00 | 1.00 | 1.00 | 3.54 | 1.00 | 1.00 | 1.00 | 1.00 | 1.00 | 1.00 | 1.03 |
| PHQ3 | 1.00 | 1.00 | 1.00 | 1.00 | 1.00 | 1.00 | 1.00 | 1.00 | 1.00 | 4.33 | 1.00 | 0.81 | 1.00 | 1.00 | 1.00 | 1.00 |
| PHQ4 | 1.00 | 1.00 | 3.44 | 1.00 | 1.00 | 1.00 | 1.00 | 1.46 | 1.00 | 1.00 | 1.00 | 0.98 | 1.00 | 1.00 | 1.00 | 1.00 |
| PHQ5 | 1.00 | 1.00 | 1.00 | 1.00 | 1.36 | 1.00 | 1.00 | 2.57 | 1.00 | 1.00 | 1.02 | 3.78 | 1.00 | 1.00 | 1.22 | 1.06 |
| PHQ6 | 1.00 | 1.00 | 1.00 | 1.00 | 1.00 | 1.00 | 1.46 | 4.01 | 3.08 | 1.00 | 2.67 | 1.00 | 6.43 | 1.00 | 1.00 | 1.00 |
| PHQ7 | 1.00 | 1.00 | 1.00 | 1.00 | 1.00 | 1.00 | 1.00 | 1.00 | 0.78 | 1.00 | 1.00 | 1.00 | 1.00 | 1.00 | 1.00 | 1.00 |
| PHQ8 | 1.00 | 1.00 | 1.00 | 1.00 | 1.33 | 1.00 | 1.00 | 1.00 | 1.00 | 1.00 | 1.00 | 1.00 | 1.00 | 1.00 | 2.60 | 1.00 |
| PHQ9 | 1.00 | 1.00 | 1.00 | 1.00 | 1.00 | 1.00 | 1.00 | 1.00 | 1.00 | 1.00 | 1.00 | 1.13 | 1.00 | 1.00 | 1.08 | 5.72 |

***Note:*** GAD, Generalized Anxiety Disorder; PHQ, Patient Health Questionnaire; T2, the COVID-19 transitional period; T3, the COVID-19 control period; COVID-19, Coronavirus disease 2019.

**Supplementary Table 9.** Weighted adjacency matrix of persistence growth group within T2→T3 network.

|  | GAD1 | GAD2 | GAD3 | GAD4 | GAD5 | GAD6 | GAD7 | PHQ1 | PHQ2 | PHQ3 | PHQ4 | PHQ5 | PHQ6 | PHQ7 | PHQ8 | PHQ9 |
| --- | --- | --- | --- | --- | --- | --- | --- | --- | --- | --- | --- | --- | --- | --- | --- | --- |
| GAD1 | 1.15 | 0.80 | 0.74 | 0.88 | 0.81 | 0.87 | 0.73 | 0.86 | 0.80 | 0.84 | 1.00 | 0.79 | 0.73 | 0.77 | 0.71 | 0.64 |
| GAD2 | 1.00 | 1.40 | 1.00 | 0.98 | 1.00 | 0.96 | 1.14 | 0.93 | 1.00 | 0.96 | 0.92 | 0.92 | 0.96 | 0.96 | 1.00 | 1.00 |
| GAD3 | 1.00 | 1.00 | 1.32 | 0.92 | 0.94 | 0.94 | 1.00 | 0.73 | 0.92 | 0.93 | 0.79 | 1.00 | 0.90 | 0.84 | 0.88 | 0.90 |
| GAD4 | 0.84 | 0.87 | 0.91 | 1.16 | 0.91 | 0.85 | 0.84 | 1.00 | 0.80 | 0.93 | 0.85 | 0.89 | 0.84 | 1.00 | 0.87 | 0.83 |
| GAD5 | 1.00 | 1.15 | 1.00 | 1.26 | 1.81 | 1.00 | 1.17 | 0.98 | 1.00 | 1.05 | 0.82 | 1.00 | 0.97 | 1.20 | 1.45 | 1.20 |
| GAD6 | 0.95 | 0.78 | 0.86 | 0.79 | 0.71 | 1.37 | 0.70 | 1.00 | 1.00 | 0.94 | 1.00 | 1.00 | 0.88 | 0.71 | 0.76 | 0.80 |
| GAD7 | 0.99 | 1.15 | 1.09 | 1.00 | 1.12 | 1.00 | 2.00 | 1.00 | 1.00 | 1.00 | 0.97 | 1.00 | 1.10 | 1.00 | 1.27 | 1.13 |
| PHQ1 | 0.91 | 0.85 | 0.89 | 0.92 | 0.88 | 1.00 | 0.75 | 2.12 | 1.08 | 1.00 | 1.40 | 1.17 | 1.13 | 1.05 | 0.93 | 0.80 |
| PHQ2 | 1.00 | 1.05 | 1.02 | 1.00 | 0.98 | 1.00 | 1.00 | 1.00 | 1.93 | 0.81 | 1.14 | 1.00 | 1.17 | 1.02 | 1.00 | 1.06 |
| PHQ3 | 1.00 | 1.02 | 1.00 | 0.96 | 1.10 | 0.90 | 1.05 | 1.00 | 1.00 | 3.16 | 1.20 | 1.23 | 0.86 | 1.03 | 1.03 | 1.08 |
| PHQ4 | 1.00 | 0.70 | 0.80 | 0.87 | 0.77 | 1.00 | 0.76 | 1.22 | 1.00 | 0.96 | 1.39 | 0.77 | 0.61 | 0.88 | 0.87 | 0.62 |
| PHQ5 | 0.91 | 0.87 | 0.94 | 0.93 | 1.01 | 0.87 | 1.03 | 1.00 | 0.89 | 1.19 | 1.00 | 2.19 | 0.81 | 0.97 | 1.06 | 1.00 |
| PHQ6 | 1.00 | 1.16 | 1.12 | 0.98 | 1.00 | 1.00 | 1.14 | 1.00 | 1.23 | 0.80 | 1.00 | 0.97 | 2.98 | 1.11 | 1.02 | 1.28 |
| PHQ7 | 1.00 | 0.93 | 0.97 | 0.91 | 1.01 | 0.86 | 0.93 | 1.25 | 1.00 | 1.04 | 1.00 | 1.00 | 1.10 | 2.21 | 1.07 | 0.87 |
| PHQ8 | 1.00 | 1.20 | 1.07 | 1.13 | 1.47 | 1.21 | 1.36 | 1.00 | 1.18 | 1.15 | 1.00 | 1.22 | 1.13 | 1.22 | 2.38 | 1.32 |
| PHQ9 | 0.84 | 1.01 | 1.00 | 0.95 | 1.04 | 0.88 | 1.39 | 0.80 | 0.84 | 1.00 | 0.71 | 0.99 | 1.15 | 0.93 | 1.19 | 4.31 |

***Note:*** GAD, Generalized Anxiety Disorder; PHQ, Patient Health Questionnaire; T2, the COVID-19 transitional period; T3, the COVID-19 control period; COVID-19, Coronavirus disease 2019.

**Supplementary Table 10.** Weighted adjacency matrix of chronic co-occurring group within T2→T3 network.

|  | GAD1 | GAD2 | GAD3 | GAD4 | GAD5 | GAD6 | GAD7 | PHQ1 | PHQ2 | PHQ3 | PHQ4 | PHQ5 | PHQ6 | PHQ7 | PHQ8 | PHQ9 |
| --- | --- | --- | --- | --- | --- | --- | --- | --- | --- | --- | --- | --- | --- | --- | --- | --- |
| GAD1 | 2.15 | 1.31 | 1.30 | 1.28 | 1.00 | 1.29 | 0.94 | 1.26 | 1.24 | 1.12 | 1.34 | 1.07 | 1.08 | 1.02 | 0.93 | 0.75 |
| GAD2 | 0.70 | 0.95 | 0.79 | 0.70 | 0.74 | 0.78 | 0.81 | 0.88 | 0.81 | 0.74 | 0.75 | 0.79 | 0.73 | 0.84 | 0.83 | 0.67 |
| GAD3 | 1.16 | 1.03 | 1.70 | 1.15 | 1.00 | 1.08 | 1.00 | 1.03 | 1.02 | 1.00 | 1.05 | 0.95 | 1.05 | 1.00 | 0.89 | 0.77 |
| GAD4 | 1.08 | 1.00 | 1.07 | 1.46 | 1.00 | 1.00 | 0.92 | 1.01 | 1.04 | 1.00 | 1.02 | 1.00 | 0.95 | 1.00 | 0.98 | 0.89 |
| GAD5 | 0.60 | 0.69 | 0.61 | 0.72 | 1.00 | 0.64 | 0.84 | 0.68 | 0.67 | 0.79 | 0.67 | 0.77 | 0.76 | 0.79 | 1.00 | 0.87 |
| GAD6 | 1.28 | 1.10 | 1.06 | 1.16 | 0.99 | 2.05 | 1.00 | 1.22 | 1.24 | 1.10 | 1.28 | 1.11 | 1.02 | 1.00 | 0.96 | 0.89 |
| GAD7 | 0.77 | 0.87 | 0.86 | 0.76 | 0.90 | 0.70 | 1.61 | 0.80 | 0.81 | 0.81 | 0.76 | 0.87 | 0.98 | 0.90 | 0.96 | 1.00 |
| PHQ1 | 1.47 | 1.32 | 1.35 | 1.41 | 1.23 | 1.34 | 1.14 | 2.69 | 1.58 | 1.49 | 1.89 | 1.50 | 1.56 | 1.80 | 1.34 | 1.00 |
| PHQ2 | 1.23 | 1.27 | 1.24 | 1.18 | 1.26 | 1.27 | 1.24 | 1.18 | 1.97 | 1.14 | 1.17 | 1.15 | 1.38 | 1.16 | 1.48 | 1.44 |
| PHQ3 | 1.08 | 1.00 | 1.05 | 1.08 | 1.00 | 1.15 | 1.00 | 1.21 | 1.14 | 2.77 | 1.35 | 1.35 | 1.02 | 1.06 | 1.06 | 1.00 |
| PHQ4 | 1.41 | 1.11 | 1.17 | 1.29 | 1.03 | 1.35 | 1.00 | 1.63 | 1.36 | 1.32 | 2.13 | 1.37 | 1.20 | 1.31 | 1.07 | 1.00 |
| PHQ5 | 0.97 | 1.00 | 1.00 | 1.00 | 1.01 | 1.02 | 1.00 | 1.14 | 1.13 | 1.25 | 1.24 | 2.27 | 1.03 | 1.13 | 1.18 | 1.00 |
| PHQ6 | 1.31 | 1.24 | 1.16 | 1.12 | 1.00 | 1.08 | 1.23 | 1.17 | 1.31 | 0.96 | 1.08 | 1.00 | 2.80 | 1.11 | 1.12 | 1.41 |
| PHQ7 | 1.17 | 1.13 | 1.23 | 1.26 | 1.35 | 1.13 | 1.05 | 1.33 | 1.18 | 1.15 | 1.31 | 1.13 | 1.08 | 2.06 | 1.17 | 1.00 |
| PHQ8 | 0.66 | 0.87 | 0.76 | 0.84 | 1.05 | 0.94 | 1.03 | 0.77 | 0.82 | 0.85 | 0.73 | 0.82 | 0.78 | 0.99 | 1.51 | 1.02 |
| PHQ9 | 0.55 | 0.81 | 0.70 | 0.60 | 0.82 | 0.64 | 0.99 | 0.46 | 0.63 | 0.65 | 0.46 | 0.59 | 0.84 | 0.60 | 0.80 | 3.30 |

***Note:*** GAD, Generalized Anxiety Disorder; PHQ, Patient Health Questionnaire; T2, the COVID-19 transitional period; T3, the COVID-19 control period; COVID-19, Coronavirus disease 2019.


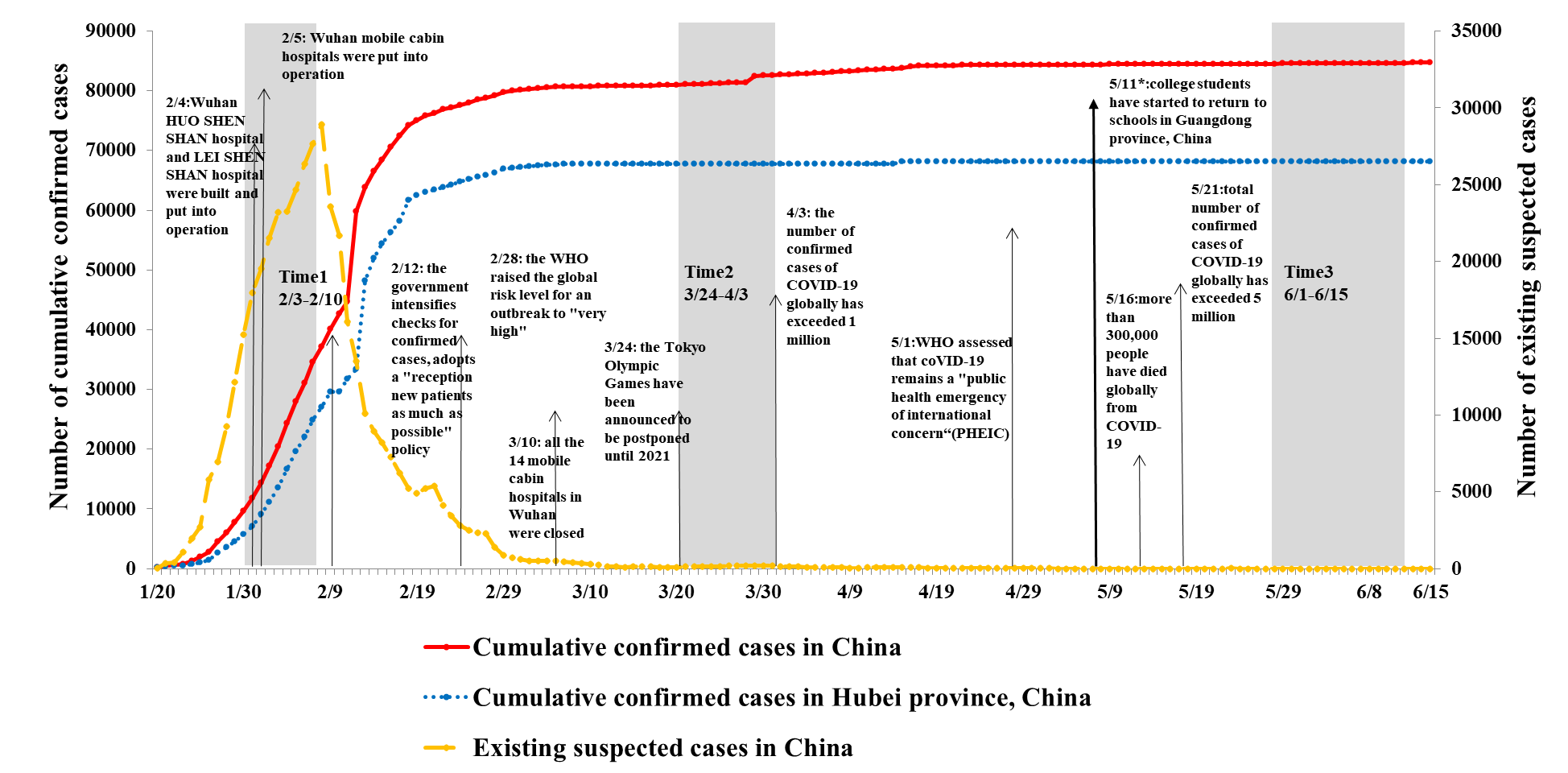


**Supplementary Figure 1.** The national pandemic trend of the 2019 coronavirus disease (COVID-19) in China and sampling times. This figure is reproduced from previous studies (Wang, Zhao, Ross, et al., 2022; Wang, Zhao, Zhai, et al., 2022).


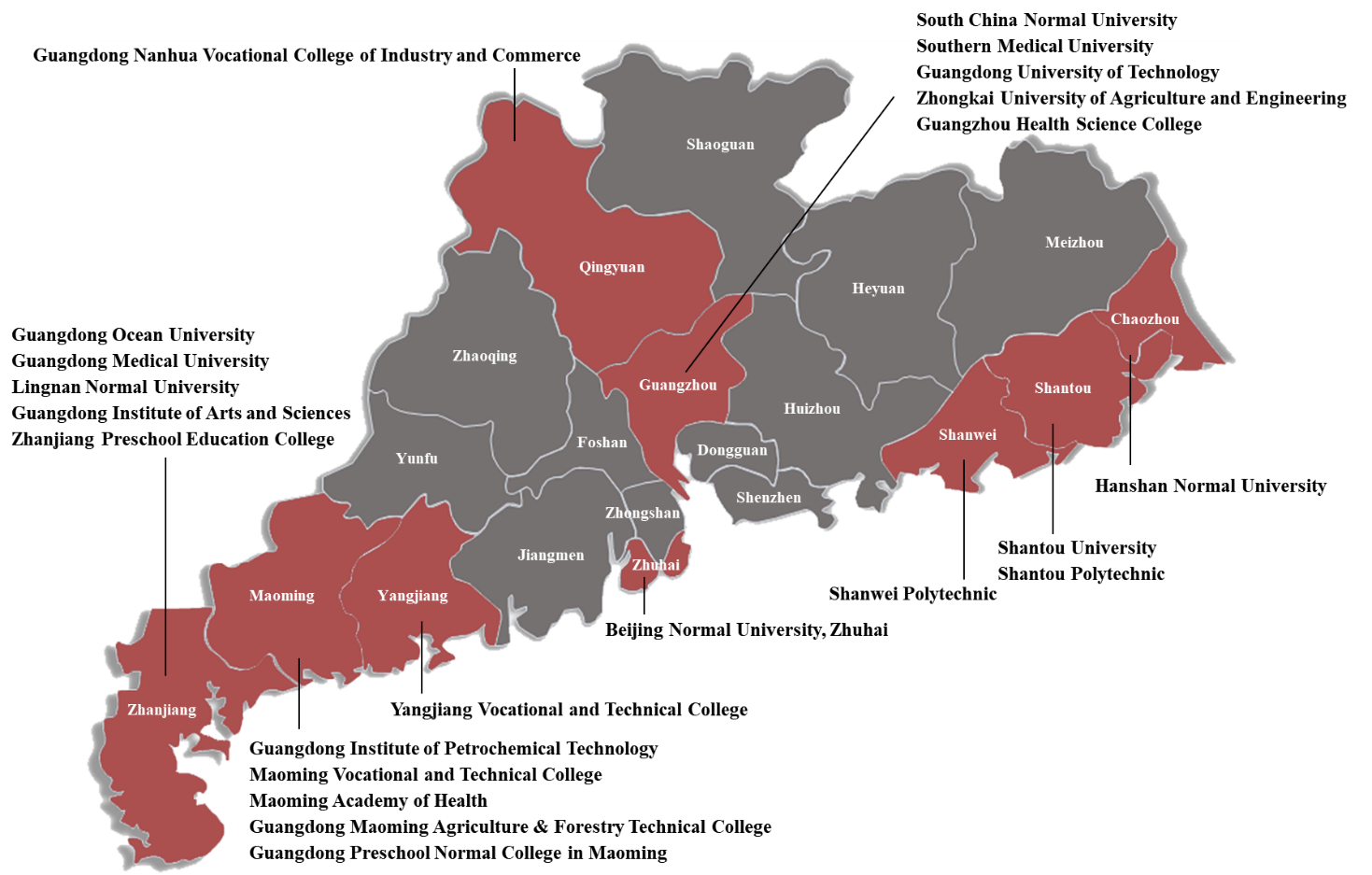


**Supplementary Figure 2.** Geographical distribution of participating colleges and universities. This figure is reproduced from previous study (Zhang et al., 2021).


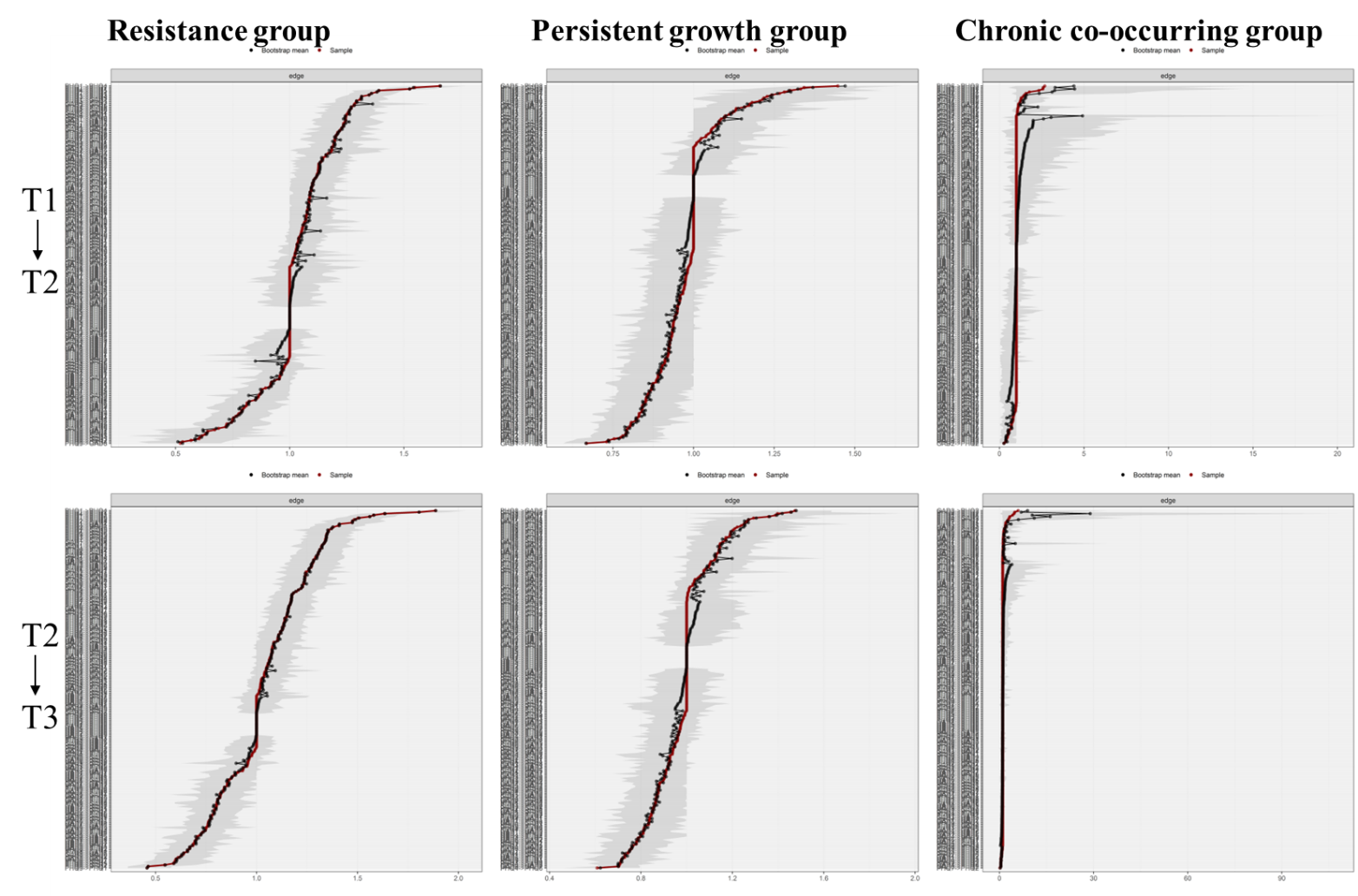


**Supplementary Figure 3.** Bootstrapped 95% confidence intervals around each edge weight across the heterogeneous co-developmental trajectories of anxiety and depressive symptoms.


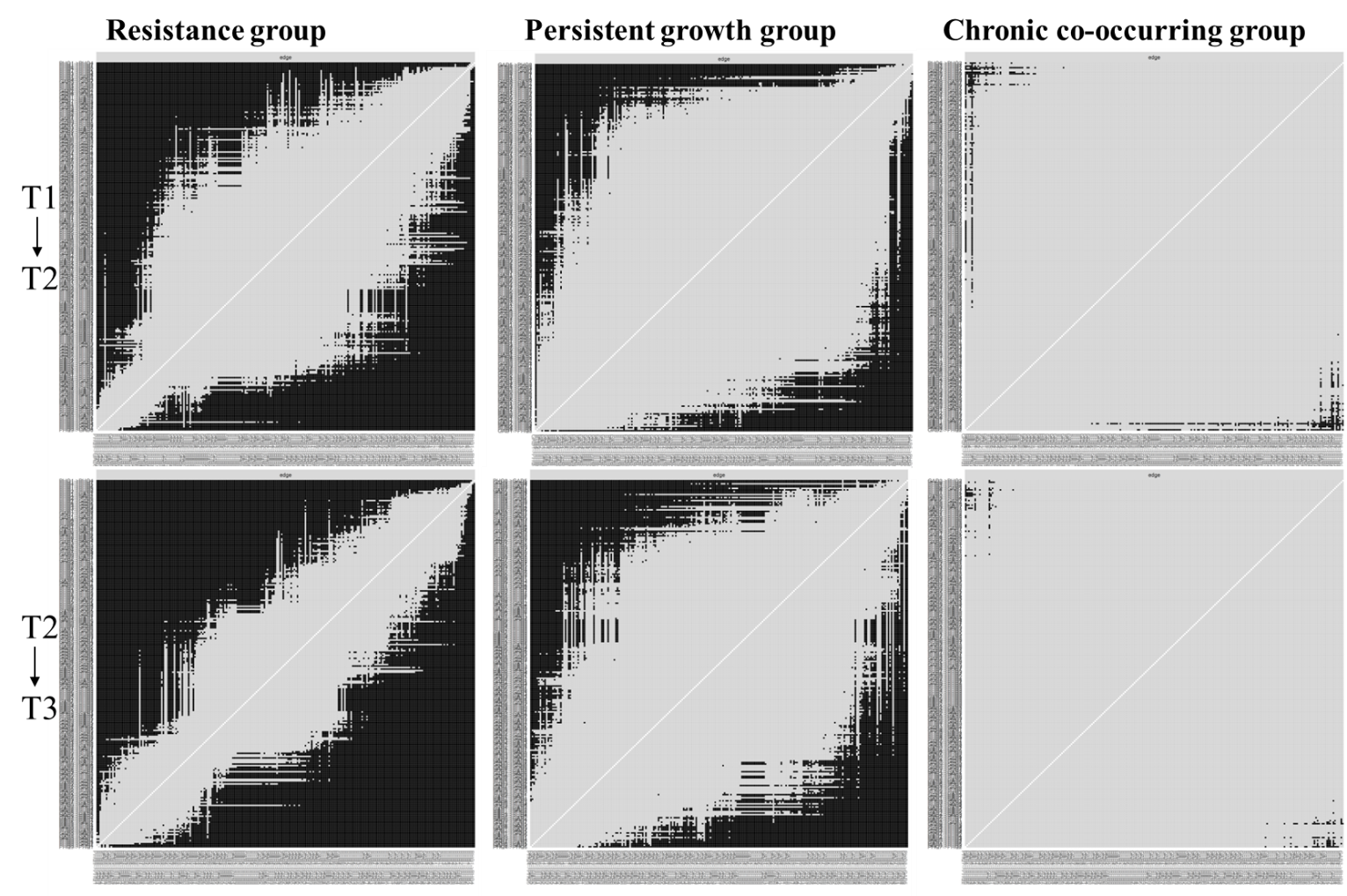


Supplementary Figure 4. Edge weight difference tests across the heterogeneous co-developmental trajectories of anxiety and depressive symptoms.

**
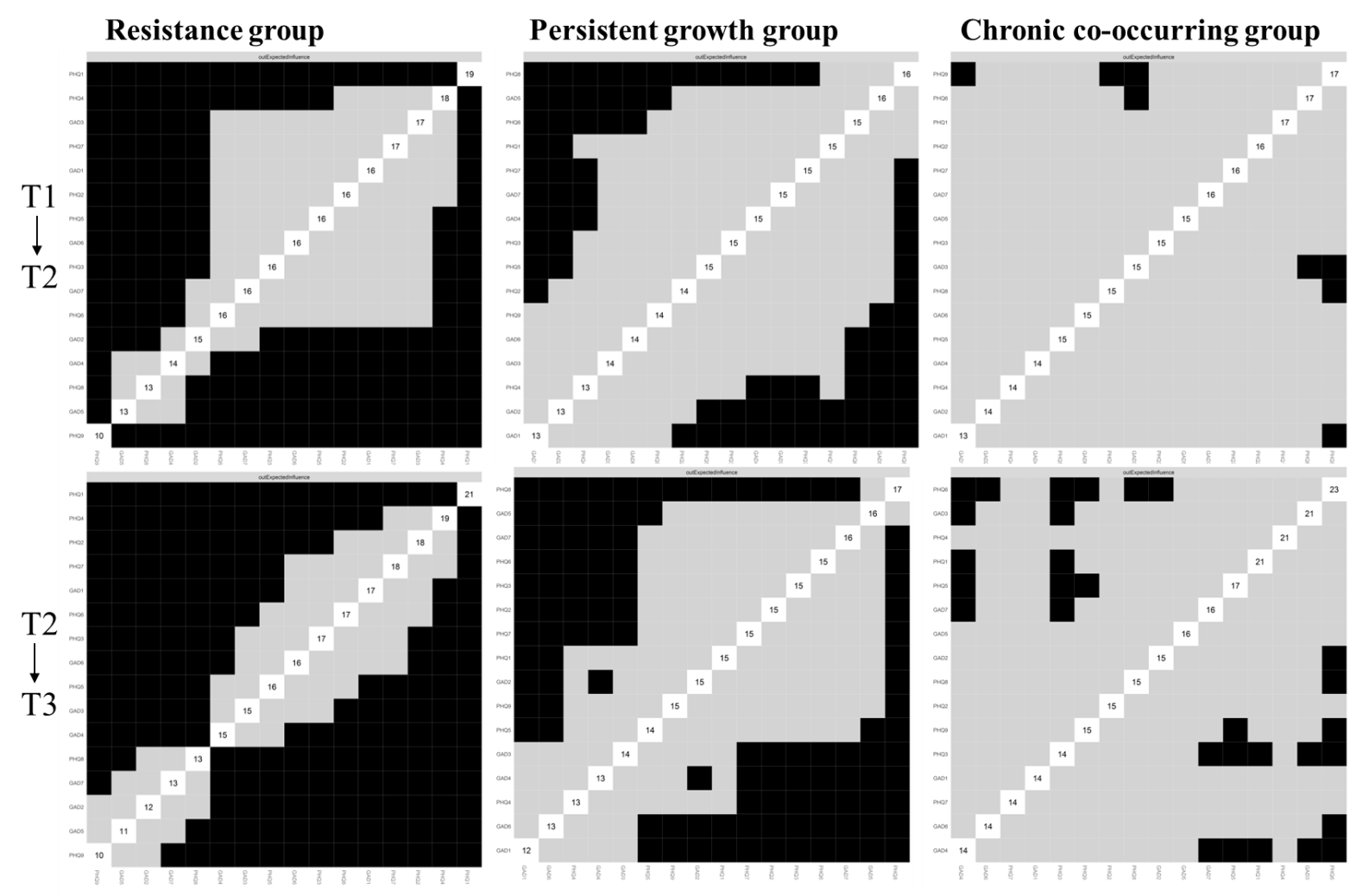
**

**Supplementary Figure 5.** Centrality difference tests of Out Expected Inﬂuence across the heterogeneous co-developmental trajectories of anxiety and depressive symptoms.


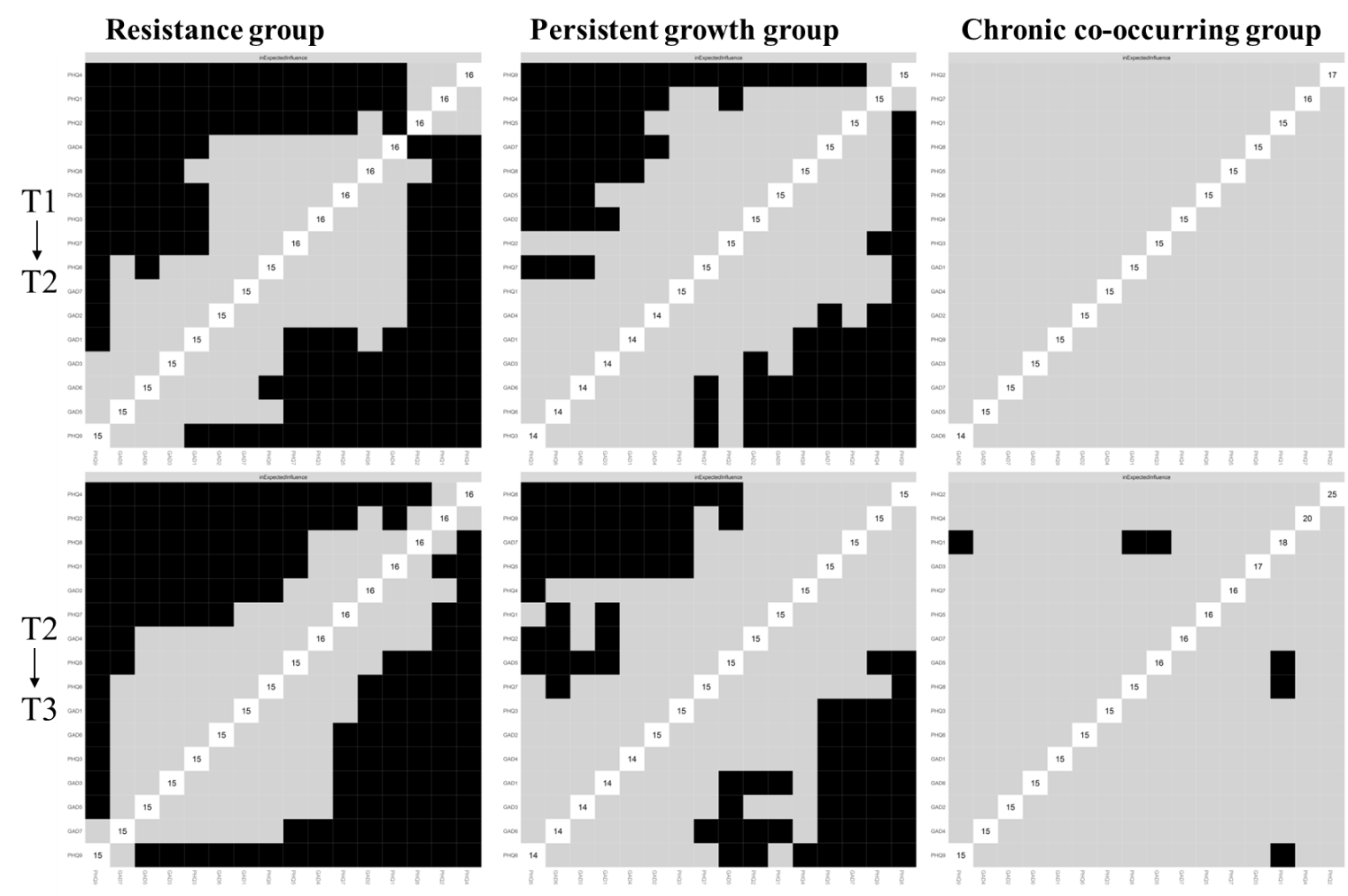


**Supplementary Figure 6.** Centrality difference tests of In Expected Inﬂuence across the heterogeneous co-developmental trajectories of anxiety and depressive symptoms.


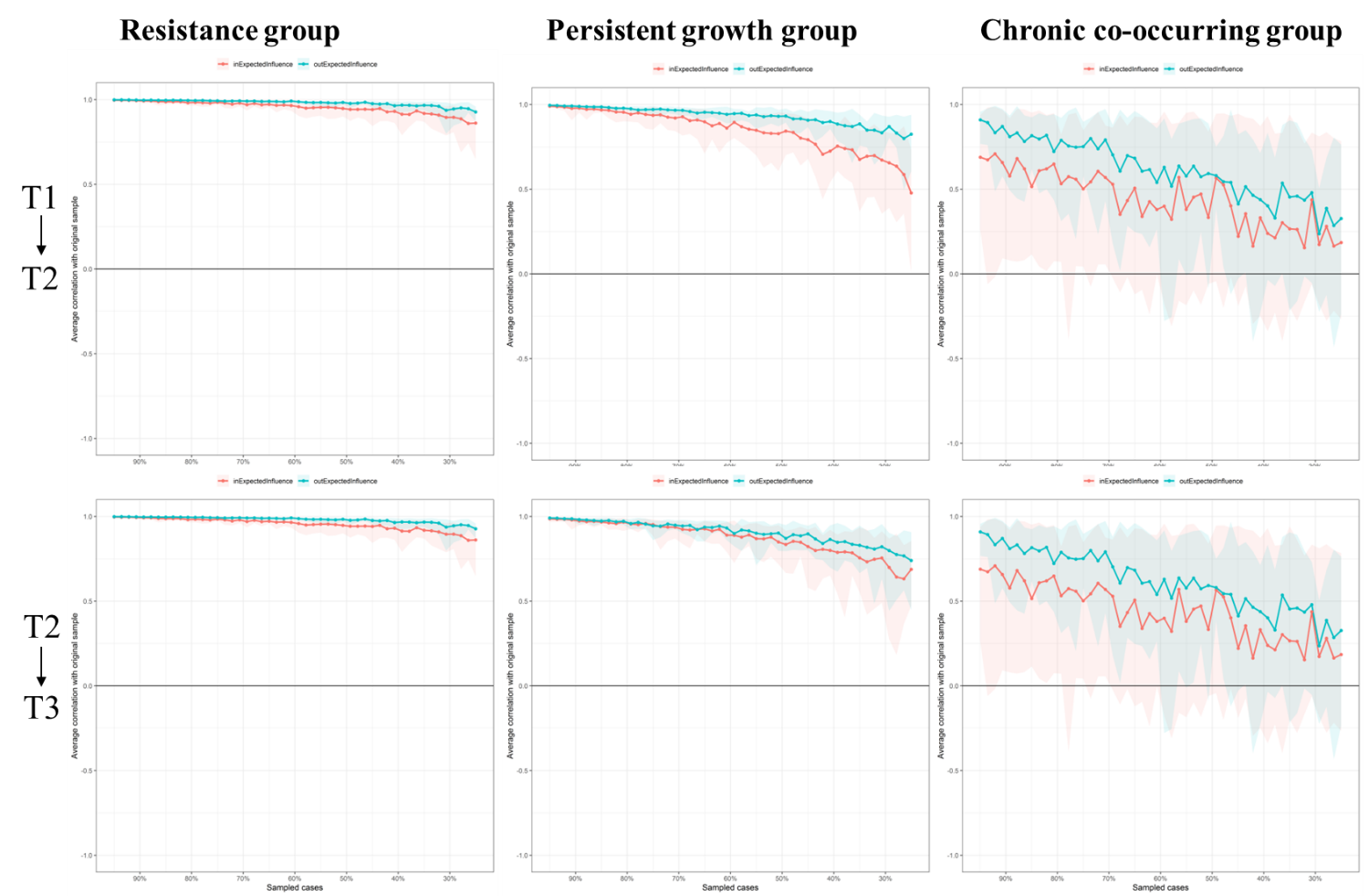


**Supplementary Figure 7.** Stability of centrality measures across the heterogeneous co-developmental trajectories of anxiety and depressive symptoms.
